# Early Versus Late Antipseudomonal β-Lactam Antibiotic Dose Adjustment in Critically Ill Sepsis Patients With Acute Kidney Injury: A Prospective Observational Cohort Study

**DOI:** 10.1093/ofid/ofae059

**Published:** 2024-02-01

**Authors:** Namareq F Aldardeer, Maram M Alshreef, Emad A Alharbi, Ahmad K Aljabri, Mohammad H Aljawadi, Thamer A Almangour, Saad Alobaili, Mohammed I Alarifi, Awad Alomari, Abdullah M Alhammad

**Affiliations:** Department of Pharmacy Services, King Faisal Specialist Hospital and Research Centre, Jeddah, Saudi Arabia; Department of Pharmacy Services, Prince Sultan Military Medical City, Riyadh, Saudi Arabia; Department of Pharmacy Services, King Fahad Hospital, Madinah, Saudi Arabia; Department of Pharmacy Services, King Fahad Hospital, Madinah, Saudi Arabia; Department of Clinical Pharmacy, College of Pharmacy, King Saud University, Riyadh, Saudi Arabia; Department of Clinical Pharmacy, College of Pharmacy, King Saud University, Riyadh, Saudi Arabia; Department of Medicine, Nephrology Unit, King Saud University, Riyadh, Saudi Arabia; Department of Critical Care Medicine, College of Medicine, King Saud University, Riyadh, Saudi Arabia; College of Medicine, Alfaisal University, Riyadh, Saudi Arabia; Department of Clinical Pharmacy, College of Pharmacy, King Saud University, Riyadh, Saudi Arabia; Corporate Department of Pharmacy Services, King Saud University Medical City, King Saud University, Riyadh, Saudi Arabia

**Keywords:** acute kidney injury, β-lactams, intensive care unit, sepsis, septic shock

## Abstract

**Background:**

Acute kidney injury (AKI) is a common complication of sepsis, contributing to an increased mortality rate. However, some studies have demonstrated that renal function improves in sepsis patients with AKI within 48 hours, raising questions about the necessity for early antibiotic adjustment. This study evaluates the association between the timing of antipseudomonal β-lactam dose adjustment and the outcomes of critically ill sepsis patients with AKI.

**Methods:**

A prospective, multicenter observational study of critically ill patients aged ≥18 years admitted to the intensive care unit with sepsis and AKI and started on antipseudomonal β-lactam therapy. After the initial dose, eligible patients were grouped as early β-lactam antibiotic (E-BLA) or late β-lactam antibiotic (L-BLA) dose adjustments based on the administration of subsequent renally adjusted doses within 24 hours and after 24 hours of sepsis recognition, respectively. The main outcome of interest was in-hospital mortality.

**Results:**

Among 1185 patients screened, 224 (mean age, 62.7 ± 16.8 years; 62% were male) met inclusion criteria. Eighty-four and 140 patients were included in the E-BLA and L-BLA groups, respectively. Approximately half of the cohort presented with AKI stage II, and piperacillin-tazobactam was prescribed as initial empirical therapy in more than 50% of the cohort. In the multivariable Cox proportional hazards model, L-BLA was associated with a significant reduction in in-hospital mortality compared to E-BLA (hazard ratio, 0.588 [95% confidence interval, .355–.974]).

**Conclusions:**

In sepsis patients with AKI, L-BLA was associated with in-hospital mortality benefits.

## Teaser

This study investigated the timing of β-lactam antibiotic dose adjustment in sepsis patients with acute kidney injury. The findings indicated that delaying antipseudomonal β-lactam antibiotic dose adjustment was associated with reduced in-hospital mortality.

Sepsis and septic shock are life-threatening medical emergencies accounting for 20% of all-cause deaths worldwide [1]. Sepsis mortality among patients treated in intensive care units (ICUs) can be as high as 40% [2]. Acute kidney injury (AKI) is a common complication of sepsis [3]. Critically ill patients with sepsis often experience early AKI, defined by AKI onset within 24 hours of hypotension initiation. This early AKI manifests in >60% of cases and is linked to a mortality rate of approximately 70% [4, 5]. Nevertheless, renal recovery was observed in about 50% of patients with sepsis and AKI within 48 hours of admission [6]. Consequently, uncertainty surrounds the need for antibiotic dose adjustment in these scenarios.

Early initiation of adequate antimicrobial therapy can reduce sepsis-related mortality [7–9]. Adequate empirical antimicrobial therapy involves the use of an agent that exhibits in vitro activity against target pathogens, administered at doses capable of reaching pharmacodynamic targets in vivo [6]. Indeed, patients with septic shock who develop AKI within 24 hours of hypotension onset are more likely to experience longer delays of 1.7 hours in receiving antimicrobial therapy than septic shock patients without AKI [4]. A meta-analysis reported increased odds of mortality associated with inadequate therapy during the first 48 hours of treatment [10]. On the other hand, the 2016 and 2021 Survival Sepsis Campaign guidelines stated that antimicrobial therapy should always be started with a total high-end-loading dose [8] and recommended optimizing antimicrobial dosage strategies based on accepted pharmacokinetic/pharmacodynamic (PK/PD) principles and specific drug properties [11]. Septic patients are in a hyperdynamic state, which may lead to increased antibiotic clearance and alterations in volume of distribution following resuscitation [8, 11].

Given the widespread use of β-lactams for empirical therapy in patients with sepsis and septic shock, the survival benefits of early appropriate antimicrobial treatment, and the PK/PD changes in sepsis patients with AKI, the decision to defer β-lactam dose reduction in sepsis patients with AKI patients remains challenging. Our hypothesis was that in sepsis patients with AKI, administration of the initial empiric β-lactam in full (not renally adjusted) doses for >24 hours after sepsis recognition would reduce mortality in comparison with the administration of full doses for <24 hours. One recent study showed a significant reduction in norepinephrine-free days among septic shock patients who received a cumulative 48-hour piperacillin-tazobactam dose of ≥27 g [12]. Nevertheless, data for the influence of β-lactam dose reduction in sepsis patients with AKI on clinical outcomes are still lacking [8, 13]. Therefore, our study aimed to evaluate the association between the timing of β-lactam antibiotic dose adjustment and the outcomes of critically ill sepsis patients with AKI.

## METHODS

### Study Design and Setting

A multicenter, prospective observational study was conducted between November 2019 and September 2021 at 4 centers in the Kingdom of Saudi Arabia (Supplementary Table 1). The study was reviewed and approved with a waiver of informed consent by the Institutional Review Board of each center. The study procedures followed the ethical standards of the institutional committee and the Helsinki Declaration of 1975.

### Population

**Figure 1.**
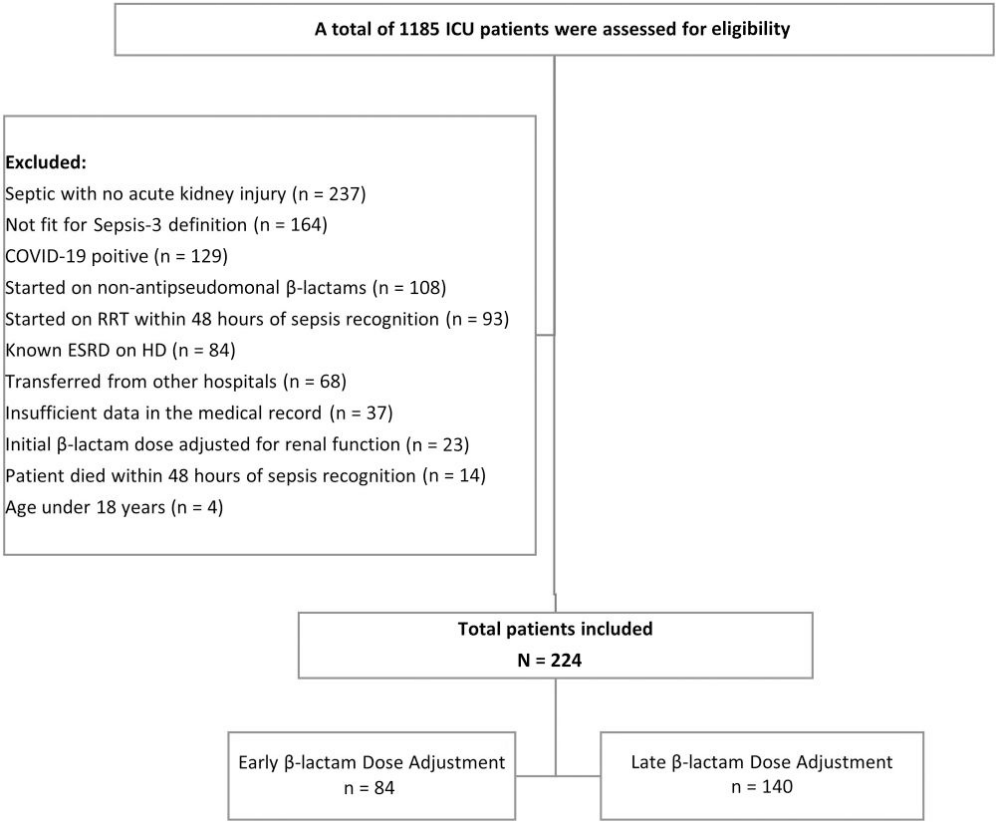
Flow diagram of patient screening. Abbreviations: COVID-19, coronavirus disease 2019; ESRD, end-stage renal disease; HD, hemodialysis; ICU, intensive care unit; RRT, renal replacement therapy.

The study population included critically ill patients with sepsis and AKI aged ≥18 years who were admitted to the ICU and received an initial full dose of antipseudomonal β-lactam antibiotics. The study excluded patients who did not meet the Sepsis-3 definition and those with unknown baseline renal function, showed no AKI upon sepsis recognition, received the initial dose of β-lactam adjusted for renal function, were started on non-antipseudomonal β-lactams, had end-stage renal disease or were receiving dialysis, were initiated on renal replacement therapy (RRT) within 48 hours of sepsis recognition, had a confirmed diagnosis of coronavirus disease 2019 (COVID-19), were transferred from outside hospitals, had insufficient data in the medical record, or died within 48 hours of sepsis recognition (Figure 1).

### Definitions

After receiving the initial full dose of antipseudomonal β-lactam upon sepsis recognition, participants were stratified into 2 groups according to the time to β-lactam renal dose reduction. The early β-lactam dose adjustment (E-BLA) group included patients who received a reduced renal dose of β-lactam within the first 24 hours of sepsis recognition, while the late β-lactam dose adjustment (L-BLA) group included patients who received a reduced renal dose of β-lactam after 24 hours from sepsis recognition. Clinical pharmacists observed the antibiotic dosing ordered by physicians for up to 24 hours after initiation without intervening. If the physician adjusted the antibiotic dose within the initial 24 hours, the clinical pharmacist recorded this patient information under the E-BLA group. Conversely, if there was no dose modification within the first 24 hours of antibiotic initiation, clinical pharmacists adjusted the renal dose after this period and collected patient information under the L-BLA group. Sepsis and septic shock were defined based according to the Sepsis-3 definition [14]. AKI was defined based on the Kidney Disease: Improving Global Outcomes (KDIGO) criteria. Stage I is an increase in serum creatinine (SCr) 1.5- to 1.9-fold from baseline, stage II is an increase in SCr 2- to 2.9-fold from baseline, and stage III is an increase in SCr 3-fold from baseline or SCr of ≥353.6 mmol/L. Transient AKI was defined as the complete reversal of AKI within 48 hours. Resolution of kidney injury was defined as a decrement of ≥50% in the serum creatinine level within 7 days of AKI onset [15, 16]. Escalation of antibiotic therapy was defined as switching patients from antibiotics with a lower spectrum of activity to antibiotics with a broader spectrum of activity [17] (Supplementary Table 2).

### Data Collection

Clinical pharmacists who participated in rounds with the team screened critically ill adult patients for eligibility. The screening was conducted 5 days weekly from 7:30 Am to 5:00 Pm. Patients included in the study at the time of sepsis recognition (Supplementary Table 2). All study-participating centers adhered to a specific renal dosing guideline for β-lactam antibiotics (Supplementary Table 3). The following information was collected from the hospital system: demographic characteristics; comorbidities; AKI stage; length of ICU and hospital stay; and SCr, lactic acid, procalcitonin, and C-reactive protein (CRP) levels. We calculated creatinine clearance (CrCl) using the kinetic glomerular filtration rate (GFR) formula using the Jelliffe equation. Data pertaining to the Acute Physiology and Chronic Health Evaluation II (APACHE-II) score during the first 24 hours after ICU admission, Sequential Organ Failure Assessment (SOFA) score during the first 24 hours after ICU admission, infection source, the medications used (antibiotics, corticosteroids, vasopressors, combined nephrotoxic), and the requirement for RRT were also collected. We used the REDCap system (Vanderbilt University, Nashville, Tennessee) to collect patient information.

### Endpoints

The primary outcome was in-hospital mortality within the first 90 days of follow-up. The secondary outcomes included transient AKI, resolution of kidney injury during the hospital stay, need for RRT, and the length of ICU and hospital stay.

### Sample Size and Statistical Analysis

No previous randomized trials have addressed the association between patient outcomes and the timing of β-lactam renal dose reduction in critically ill patients with sepsis and AKI. The results of an earlier study indicated that the mortality rates associated with antibiotic dose adjustment among ICU patients with renal impairment were higher than those associated with a usual dose (74.1% vs 55.5%) [13]. We hypothesized that the L-BLA group (intervention) would show a 20% lower absolute risk of the primary outcome than the E-BLA group (control) and that a sample size of 224 patients would be required for the study to have 80% power to detect this difference in a 2-sided test at a 5% significance level.

Categorical variables were described as frequency and percentage values, while continuous variables were described using mean ± standard deviation or median and interquartile range. We used the χ^2^ or Fisher exact test to compare categorical variables and an independent *t* test to compare continuous variables; the Mann-Whitney *U* test was performed to compare nonnormally distributed variables.

A multivariable Cox proportional hazards model was used to estimate the effects of β-lactam dose reduction on in-hospital mortality. Covariate selection was based on the authors’ perceived clinical relevance, as well as prior studies [7, 10]. Hazard ratios (HRs) with 95% confidence intervals (CIs) were obtained using maximum partial likelihood estimation. Alpha was set at *P* < .05 to define statistical significance. The log-log survivor functions of E-BLA and L-BLA for mortality were tested to assess the proportional hazard assumptions. Adjusted Kaplan-Meier survival curves were plotted following the multivariable Cox proportional hazard regressions to depict the impact of dose adjustment on mortality. For depicting the adjusted Kaplan-Meier survival curves, categorical variables were set as zeros while continuous variables were set as the means. All statistical analyses were performed using Stata version 14 (StataCorp LP, College Station, Texas) [18] and SAS 9.4 (SAS Institute, Cary, North Carolina) software [19].

Concerning model fitting, Harrell C concordance statistic was used to determine the percentage of concordance between the predicted values and the outcome pairs. In addition, the time-dependent area under the curve (AUC) that plots the receiver operating characteristic (ROC) curve at different time points with 95% CI was used. The latter uses the inverse probability of censoring weighting to calculate the time-dependent ROC. Last, Cox-Snell residuals were predicted following the multivariable Cox regression and used as survival times to estimate the cumulative hazard of these residuals using the Nelson-Aalen approach. Next, the residuals were plotted against the estimated cumulative hazards. If the Cox regression model was fit, these residuals should follow an exponential distribution with mean of 1, creating a roughly straight line.

In a separate sensitivity analysis, the inverse probability of treatment weighting (IPTW) approach was used to mitigate residual confounding [20]. A multivariable logistic regression with forward selection was initially used to determine the best model to predict the treatment assignment. A *P* value of .1 was used as an entry criterion. Subsequently, the identified variables from the model were used in the PSMATCH procedure within SAS software to produce propensity scores. The scores were used to construct the inverse probability of treatment weights. Last, the Cox multivariable regression model was weighted by the constructed weights.

To mitigate potential immortal time bias [21], which may arise if the time to mortality was calculated upon ICU admission, we calculated the times to mortality from the beginning of the 49th hour until either mortality occurred or censoring took place. This action ensures that all patients may experience the outcome during the follow-up period. Patients were censored upon discharge if they were alive.

## RESULTS

### Characteristics of the Study Population

We screened 1185 patients for eligibility and included 224 patients (mean age, 62.7 ± 16.8 years; males, 62.1%), including 84 patients in the E-BLA group and 140 in the L-BLA group (Figure 1). Most of the included patients were admitted to medical ICUs (171 patients [76.3%]) with median APACHE-II and SOFA scores of 23 and 9, respectively. Pneumonia was the most documented source of infection (114 patients [50.9%]). Piperacillin-tazobactam was prescribed as initial empirical therapy in 123 (54.9%) patients. Based on the KDIGO criteria, approximately half of the included patients (110 [49.1%]) presented with AKI stage II. There were statistically significant intergroup differences observed in the baseline characteristics, including higher median APACHE-II score, age, and serum creatinine concentration (μmol/L) at inclusion, 24 and 48 hours in the E-BLA group. However, patients in the L-BLA group had a higher prevalence of respiratory infection compared to those in the E-BLA group (Table 1).

**Table 1.** Demographics and Clinical Characteristics of the Study Participants

Variable	All Patients	Early Antibiotic Dose Adjustment (E-BLA)	Late Antibiotic Dose Adjustment (L-BLA)	*P* Value
	(N = 224)	(n = 84)	(n = 140)	
Age, y, mean ± SD	62.7 ± 16.8	66.9 ± 14.6	60.1 ± 16.9	.002
Male sex	139 (62.1)	47 (56)	92 (65.7)	.1
Weight, kg, mean ± SD	75.6 ± 20.4	73.7 ± 17.6	76.7 ± 21.9	.3
ICU admission type				.4
Medical	171 (76.3)	62 (73.8)	109 (77.9)	
Surgical	53 (23.7)	22 (26.2)	31 (22.1)	
APACHE-II score^a^, median (IQR)	23 (19–28)	25 (19.5–29.5)	23 (18.5–27)	.02
SOFA score^a^, median (IQR)	9 (7–11)	10 (7–12)	9 (7–11)	.07
Corticosteroid use	141 (62.9)	47 (56)	94 (67.1)	.09
Vasopressor use	194 (86.6)	77 (91.7)	117 (83.6)	.09
Lactic acid, µmol/L, mean ± SD	4 ± 3.2	4.4 ± 3	3.7 ± 3.4	.09
Procalcitonin, ng/mL, mean ± SD	1.6 ± 0.6	1.6 ± 0.6	1.7 ± 0.6	.3
CRP, nmol/L, mean ± SD	156.1 ± 121.1	167.8 ± 158.4	151.5 ± 104	.5
Comorbidities				
Cardiovascular diseases	160 (71.4)	66 (78.6)	94 (67.1)	.06
Diabetes	144 (64.3)	57 (67.9)	87 (62.1)	.3
Nonhematologic malignancy	40 (17.9)	16 (19)	24 (17.1)	.7
Chronic kidney disease	23 (10.3)	13 (15.5)	10 (7.1)	.05
Liver disease	11 (4.9)	6 (7.1)	5 (3.6)	.2
Hematologic malignancy	9 (4)	4 (4.8)	5 (3.6)	.6
Autoimmune disease	7 (3.1)	2 (2.4)	5 (3.6)	.6
Solid organ transplantation	6 (2.7)	1 (1.2)	5 (3.6)	.2
Active bleeding	4 (1.8)	2 (2.4)	2 (1.4)	.3
Source of infection				
Respiratory	114 (50.9)	32 (38.1)	82 (58.5)	.003
Intrabdominal	49 (21.9)	24 (28.6)	25 (17.9)	.06
Urinary tract	42 (18.8)	17 (20.2)	25 (17.9)	.7
Skin and soft tissue	21 (9.4)	11 (13.1)	10 (7.1)	.1
Cultures				.2
Positive	132 (58.9)	54 (64.3)	78 (55.7)	
Gram-negative organism	116 (87.8)	48 (88.8)	68 (87.1)	
Gram-positive organism	16 (12.1)	6 (11.1)	10 (12.8)	
Negative	92 (41.1)	30 (35.7)	62 (44.3)	
Escalation of therapy needed to target culture in patients with gram-negative isolates	38 (16.9)	12 (14.3)	26 (18.6)	.4
Serum creatinine, µmol/L, median (IQR)				
Baseline	71.5 (56–90)	74.5 (59.5–95.5)	72 (53.5–86)	.08
Upon inclusion	180 (138.5–242)	217 (152–281.5)	166 (127.5–214.5)	.001
24 h of inclusion	172.5 (120–230)	197.5 (140.5–264.5)	153 (113–215)	.001
48 h of inclusion	148 (95.5–238.5)	182 (123.5–293.5)	132.5 (90–209.5)	.001
AKI stage per KDIGO				.1
Stage 1	48 (21.4)	14 (16.7)	34 (24.3)	
Stage 2	110 (49.1)	39 (46.4)	71 (50.7)	
Stage 3	66 (29.5)	31 (36.9)	35 (25)	
Estimated CrCl^b^, mL/min, mean ± SD				
At 24 h	31.5 + 18.9	24.9 ± 15.3	35.2 ± 19.8	.001
At 48 h	37.3 ± 25	28.9 ± 20.3	42.7 ± 26.1	.001
Nephrotoxic combination				
Within 48 h of inclusion	179 (79.9)	68 (81)	111 (79.3)	.8
>48 h of inclusion	18 (8)	4 (4.8)	17 (10)	.2
No nephrotoxic combination	28 (12.5)	12 (14.3)	16 (11.4)	.5
Nephrotoxic agents used				
Vancomycin	171 (76.3)	62 (73.8)	109 (77.9)	.5
Aminoglycosides	2 (0.9)	0 (0)	2 (1.4)	.3
Other	4 (1.8)	4 (4.8)	0 (0)	.009
First antipseudomonal β-lactam administered				.3
Piperacillin-tazobactam	123 (54.9)	43 (51.2)	80 (57.1)	
Meropenem	78 (34.8)	34 (40.5)	44 (31.4)	
Imipenem-cilastatin	18 (8)	7 (8.3)	11 (7.9)	
Ceftazidime	3 (1.3)	0 (0)	3 (2.1)	
Cefepime	2 (0.9)	0 (0)	2 (1.4)	
Time to first β-lactam administration from sepsis recognition, min, median (IQR)	120.5 (66–138)	120 (66–150)	121 (65.5–131)	.8

Data are presented as No. (%) unless otherwise indicated.

Abbreviations: AKI, acute kidney injury; APACHE-II, Acute Physiology and Chronic Health Evaluation Assessment; CrCl, creatinine clearance; CRP, C-reactive protein; E-BLA, early β-lactam antibiotic; KDIGO, Kidney Disease: Improving Global Outcomes; ICU, intensive care unit; IQR, interquartile range; L-BLA, late β-lactam antibiotic; SD, standard deviation; SOFA, Sequential Organ Failure Assessment.

^a^APACHE-II and SOFA scores were calculated during the first 24 h after ICU admission.

^b^CrCl estimation based on Jelliffe equation.

### Mortality Outcomes

**Figure 2.**
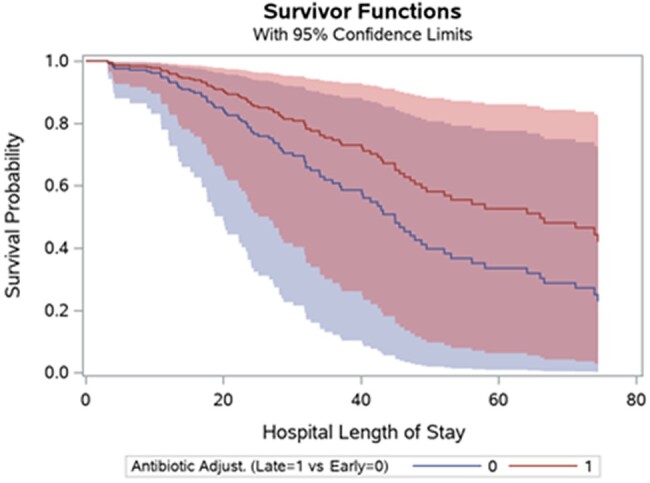
Kaplan-Meier survival analysis curve for in-hospital mortality in critically ill patients with sepsis and acute kidney injury based on the timing of dose adjustment.

After covariate adjustments, the multivariable Cox proportional hazard model showed that L-BLA was associated with a significant reduction in in-hospital mortality compared to E-BLA (HR, 0.588 [95% CI, .355–.974]) (Table 2, Figure 2). The results were consistent even after adjusting the model by the presence of gram-positive organisms (HR, 0.564 [95% CI, .340–.934]) or excluding patients with gram-positive infection from the model (HR, 0.512 [95% CI, .302–.867]). In addition, the multivariable Cox regression model with IPTW showed very consistent results (HR, 0.574 [95% CI, .3552–.923]). The results of the multivariable logistic regression for treatment assignment and the covariates’ balance before and after weighting were provided in the Supplementary Material A and B.

**Table 2.** Multivariable Cox Proportional Hazards Regression Model of In-Hospital Mortality Comparing Early Versus Late Time to β-Lactam Dose Adjustment (N = 224)

Variable	Main Model		Main Model After Adjusting by Patients With Gram-Positive Culture		Main Model After Excluding Patients With Gram-Positive Culture	
	(N = 224)		(n = 224)		(n = 208)	
	HR	(95% CI)	HR	(95% CI)	HR	(95% CI)
Late vs early β-lactam adjustment	0.588*	(.355–.974)	0.564*	(.340–.934)	0.512*	(.302–.867)
Age	1.007	(.991–1.023)	1.007	(.991–1.023)	1.006	(.990–1.022)
Female vs male sex	0.62	(.379–1.014)	0.564*	(.341–.933)	0.552*	(.330–.925)
APACHE-II score	1.042*	(1.000–1.085)	1.048*	(1.006–1.092)	1.059**	(1.014–1.105)
SOFA score	1.122*	(1.011–1.245)	1.110*	(1.000–1.232)	1.083	(.974–1.204)
Corticosteroid use	1.78	(.994–3.187)	2.009*	(1.106–3.650)	2.029*	(1.104–3.729)
Vasopressor use	0.568	(.195–1.651)	0.552	(.191–1.599)	0.567	(.194–1.653)
Lactic acid level	1.048	(.982–1.119)	1.047	(.977–1.121)	1.026	(.948–1.110)
Time to first β-lactam administration	0.999	(.996–1.002)	0.999	(.996–1.002)	0.999	(.996–1.002)
Use of any nephrotoxic drug	0.951	(.404–2.239)	1.008	(.429–2.368)	1.041	(.441–2.456)
AKI stage per KDIGO						
Stage 1 (Reference)	1	(1–1)	1	(1–1)	1	(1–1)
Stage 2	0.794	(.403–1.563)	0.701	(.353–1.391)	0.623	(.306–1.270)
Stage 3	1.298	(.629–2.680)	1.149	(.546–2.418)	0.965	(.432–2.156)
RRT	0.88	(.507–1.530)	0.899	(.512–1.579)	1.03	(.570–1.864)
Comorbidities						
Hematologic malignancy	1.534	(.465–5.063)	1.433	(.437–4.694)	1.398	(.431–4.532)
Nonhematologic malignancy	2.821***	(1.602–4.967)	2.813***	(1.601–4.944)	2.761***	(1.546–4.934)
Solid organ transplantation	0.636	(.147–2.761)	0.57	(.128–2.543)	0.616	(.133–2.850)
Autoimmune diseases	3.303	(.776–14.05)	3.3	(.792–13.76)	3.055	(.747–12.50)
Cardiovascular diseases	1.263	(.733–2.177)	1.19	(.687–2.059)	1.254	(.711–2.213)
Chronic kidney disease	1.621	(.686–3.831)	1.633	(.694–3.845)	1.701	(.727–3.982)
Source of infection						
Respiratory	1.247	(.626–2.484)	1.403	(.697–2.826)	1.255	(.611–2.578)
Intrabdominal	0.718	(.331–1.559)	0.762	(.354–1.642)	0.73	(.334–1.597)
Urinary tract	0.943	(.437–2.034)	0.931	(.436–1.986)	0.913	(.427–1.952)
Skin and soft tissue	1.547	(.645–3.715)	1.641	(.686–3.924)	1.397	(.566–3.448)
Escalation of therapy needed to target culture in patients with gram-negative isolates	0.747	(.395–1.413)	0.665	(.347–1.275)	0.659	(.342–1.271)
Gram-positive organism	…	…	0.456	(.170–1.224)	…	…

Abbreviations: AKI, acute kidney injury; APACHE-II, Acute Physiology and Chronic Health Evaluation; CI, confidence interval; HR, hazard ratio; KDIGO, Kidney Disease: Improving Global Outcomes; RRT, renal replacement therapy; SOFA, Sequential Organ Failure Assessment.

*P < .05, **P < .01, ***P < .001.

### Model Fit

Concerning model fitting, Harrell C concordance statistic for the main model was 0.7369 (ie, 73.69% concordance). In addition, the overall integrated time-dependent AUC was 0.7787 (ie, 77.87%), indicating good data. Supplementary Figure 1 shows the ROC at different time points with 95% CI around the line. Last, Supplementary Figure 2 shows a roughly straight line when the residuals were plotted against the estimated cumulative hazards, indicating a good fit for the data.

### Secondary Outcomes

In comparison with the E-BLA group, the L-BLA group showed a higher rate of 48 hours AKI recovery (70.7% vs 47.6%, *P* = .001) and resolution of kidney injury (83.6% vs 67.9%, *P* = .006). The 2 groups showed no differences in terms of the need for RRT during ICU admission (17.1% vs 21.4%, *P* = .43). The lengths of stay in the ICU and hospital were significantly higher in the L-BLA group (Table 3).

**Table 3.** Secondary Outcomes

Variable	All Patients	Early β-Lactam Dose Adjustment	Late β-Lactam Dose Adjustment	*P* Value
	(N = 224)	(n = 84)	(n = 140)	
Transient AKI, No. (%)	139 (62.1)	40 (47.6)	99 (70.7)	.001
Resolution of kidney injury, No. (%)	174 (77.7)	57 (67.9)	117 (83.6)	.006
Need for RRT, No. (%)	42 (18.8)	18 (21.4)	24 (17.1)	.43
Length of stay, d, mean ± SD				
ICU	17.2 ± 15.1	12.7 ± 12.4	20 ± 16	.001
Hospital	36.2 ± 32.8	26.4 ± 18.6	42.1 ± 37.8	.001

Abbreviations: AKI, acute kidney injury; ICU, intensive care unit; RRT, renal replacement therapy; SD, standard deviation.

## DISCUSSION

This prospective, multicenter observational study aimed to assess the association between the timing of antipseudomonal β-lactam antibiotic dose adjustment and patient outcomes in critically ill patients with sepsis and AKI. To our knowledge, this is the first study to compare the clinical outcomes of early versus late antipseudomonal β-lactams dose adjustment in patients with sepsis and AKI. In the multivariate regression analysis, we observed a statistically significant difference in-hospital mortality, favoring the L-BLA group. The increased survival rate in the L-BLA group may account for the prolonged ICU and hospital stay observed within that cohort. Furthermore, when comparing patients in the E-BLA group with those in the L-BLA group, it was observed that a higher proportion of patients in the L-BLA group experienced complete reversal of AKI within 48 hours (indicative of transient AKI) and resolution of kidney injury. This outcome could be attributed to the lower severity of illness observed at baseline in the L-BLA group.

Critically ill patients admitted with sepsis and AKI are likely to have a higher risk of hospital mortality [22, 23]. Several studies have described the link between inappropriate initial antimicrobial therapy and mortality in sepsis patients admitted to the ICU [24–26]. Inadequate antimicrobial dosing in critically ill patients is common and is a significant independent risk factor for mortality [3, 24]. Additionally, the presence of bacteria with high minimum inhibitory concentrations in the ICU that are not appropriately treated by the prescribed antimicrobial regimen is a common reason for treatment failure [27, 28]. Thus, one of the key drivers of survival benefits in patients with sepsis and septic shock is the initiation of adequate antimicrobial therapy.

In a recent study of 126 critically ill patients with renal impairment, adjusting antibiotic doses based on estimated GFR was associated with higher rates of treatment failure and mortality compared to nonadjusted doses [13]. Despite variations in patient profiles, antibiotics used, and equations applied (Cockcroft-Gault or Modification of Diet in Renal Disease), the study did not specify dose adjustment timing. Nevertheless, our results were consistent with the findings of that study and showed worsening clinical outcomes in critically ill patients who received adjusted antibiotic doses. Moreover, another multicenter retrospective study of 1279 patients with septic shock aimed to determine the influence of cumulative 48-hour piperacillin-tazobactam dosing in the early phase of septic shock. The study showed that patients who received a higher cumulative 48-hour dose had more norepinephrine-free days compared to those who received a lower dose (23.9 vs 13.6 days, *P* = .021). However, the study evaluated the outcomes only for piperacillin-tazobactam, whereas we included all antipseudomonal β-lactam antibiotics, thereby confirming the importance of avoiding early dose reduction for other β-lactams. Moreover, we counted the time to β-lactam dose adjustment from the time of sepsis recognition, which is an important measure for clinical outcomes in septic shock patients as per the Survival Sepsis Campaign guideline [11]. In conclusion, our study aligns with these studies, supporting that administering full doses of β-lactams in the early phase of septic shock in the presence of AKI is not harmful and may even be beneficial.

Antibiotic dosing in critically ill patients with unstable renal function is a topic of debate. In a survey-based study, critical care pharmacists tended to adjust the β-lactam dosage after 24 hours of therapy based on an estimated CrCl of <30 mL/minute. In contrast, infectious disease pharmacists were more likely to adjust it immediately after the first dose in the same patient population [29]. There are many equations used to estimate GFR in patients with unstable renal function; however, discordance in drug dosing among this equation has been noted [30]. Thus, the most appropriate equation to estimate renal function in patients with AKI is yet not defined. Indeed, a recent study in patients with shock reported similarities in the performance of some kinetic and static GFR equations [31].

Sepsis is a known contributing factor to AKI. However, other risk factors can increase the likelihood of AKI and delay recovery, including age, nonrenal organ failure, and disease severity [32, 33]. Additionally, the association between AKI severity and renal recovery has already been established [34]. A study of 1753 patients that compared critically ill sepsis patients with AKI and nonsepsis patients with AKI found a trend toward higher rates of renal recovery in sepsis with AKI patients [27]. In our cohort, approximately half of the patients showed AKI stage II, and almost two-thirds showed renal recovery within 48 hours. These findings were observed more often in patients in the L-BLA group, who were younger, had lower APACHE-II scores, and had higher CrCl at inclusion compared to those in the E-BLA group. Our study's findings underscore the importance of carefully customizing therapeutic strategies for patients with AKI within the initial 48 hours of septic shock recognition. Clinicians should pay close attention to individuals who may exhibit a potential for renal recovery, suggesting a possible advantage in delaying adjustments to antipseudomonal β-lactam doses during the first 48 hours following the recognition of septic shock. Since sepsis-associated AKI is often transient, we should therefore be cautious to not aggressively dose-reduce antibiotics. While factors such as severity score and AKI severity can be considered in the assessment [35], additional research in this realm is warranted.

The major strengths of our study include its multicenter nature, the prospective inclusion of a specific population (sepsis with AKI), and the initial administration of the full antipseudomonal β-lactam dose to compare the impact of 2 β-lactam dosage-reduction strategies in the first 48 hours after sepsis recognition (early and late). We also evaluated certain outcomes of interest in these populations, including transient AKI, resolution of kidney injury, and the need for RRT.

Our study also had some notable limitations. We did not evaluate the patients’ fluid resuscitation condition and its influence on outcomes such as kidney injury and mortality. Although adequate fluid resuscitation is crucial for the stabilization of patients with sepsis and septic shock [11], some randomized clinical trials suggest that early fluid resuscitation may be harmful [36]. Additionally, β-lactam administration in our study was performed by intermittent infusion. Although some benefits of extended and continuous infusion of some β-lactams (piperacillin-tazobactam) have been reported previously [37], similar findings were not obtained for other β-lactams (carbapenems) [38]. Our cohort included patients who received different initial antipseudomonal β-lactam-based regimens, of which piperacillin-tazobactam and meropenem constituted >80%. Another limitation is our lack of β-lactam monitoring services, which means we cannot be sure that toxicity (seizures, decreased cell counts, or worse, AKI) is not caused by β-lactam accumulation. However, we followed the patients’ records during ICU stay, and no side effects were reported. In addition, we did not include patients who started on non-antipseudomonal agents, nor did we investigate patient source control data. Moreover, we did not collect information on the type of corticosteroids and vasopressors used and their doses. Although there is a recent study on improved survival rates when prioritizing the administration of β-lactam over vancomycin [39], we did not investigate the order of antibiotic administration in our cohort. On the other hand, the renal recovery outcome in our study could be prone to selection bias due to the differences in the baseline characteristics in both groups, which could hinder us from giving a definite conclusion on the relation between the timing of antibiotic dose adjustment and renal recovery. Moreover, patients in the L-BLA group had more transient AKI with a higher length of stay, which could be explained by the lower severity of illness of this group at baseline leading to lower mortality and longer ICU and hospital length of stay. Thus, the result of the association between the timing of β-lactam dose adjustment and secondary patient outcomes could be affected by the presence of some group differences at baseline. Additionally, we did not investigate the impact of nephrotoxic agent dosage and frequency on outcomes, including temporary AKI and kidney injury resolution [40].

## CONCLUSIONS

Our study found a decrease in in-hospital mortality among patients with sepsis and AKI when delaying β-lactam antibiotic adjustment beyond 24 hours of sepsis recognition. This suggests potential benefits associated with a nonaggressive β-lactam dose reduction in the early phase of septic shock with AKI. Further research is needed to validate the connection between antibiotic dosing, time to kidney recovery, and patient outcomes in individuals with sepsis and AKI, providing insights into both short- and long-term outcomes.

## Supplementary Data

Supplementary materials are available at *Open Forum Infectious Diseases* online. Consisting of data provided by the authors to benefit the reader, the posted materials are not copyedited and are the sole responsibility of the authors, so questions or comments should be addressed to the corresponding author.

## Supplementary Material

ofae059_Supplementary_Data

## Notes

***Author contributions.*** All authors made substantial contributions to the conception and design, acquisition of data, analysis, and interpretation of data, and/or took part in drafting the article or revising it critically for important intellectual content. All authors agreed to submit it to the current journal, gave final approval for the version to be published, and agreed to be accountable for all aspects of the work.

***Acknowledgments*.** The authors acknowledge the Researchers Supporting Project (number RSPD2024R919), King Saud University, Riyadh, Saudi Arabia.

***Patient consent.*** The participating centers were King Saud University Medical City–Riyadh (E-20-4595), which was approved in 2020; King Fahad Hospital–Medina (5/7/2020) in 2020; Prince Sultan Military Medical City–Riyadh (HP-01-R079) in 2020; and King Faisal Specialist Hospital & Research Centre–Jeddah (2021-44) in 2021. All centers received a waiver of patient consent.

***Data availability.*** The dataset supporting the conclusions of this article is available by request from the corresponding author.
