# Supplementary material for: Early Versus Late Antipseudomonal β-Lactam Antibiotic Dose Adjustment in Critically Ill Sepsis Patients With Acute Kidney Injury: A Prospective Observational Cohort Study
Source: Open Forum Infect Dis. 2024 Feb 1;11(3):ofae059. doi: 10.1093/ofid/ofae059 (PMC10906704; doi:10.1093/ofid/ofae059)
Supplement: ofae059_Supplementary_Data [file ofae059_supplementary_data.zip › ADDITI~1.DOC]

| **Model Information** | | |
| --- | --- | --- |
| **Data Set** | WORK.TOSAS1 |  |
| **Response Variable** | late_ab | late_ab |
| **Number of Response Levels** | 2 |  |
| **Model** | binary logit |  |
| **Optimization Technique** | Fisher's scoring |  |

| **Number of Observations Read** | 224 |
| --- | --- |
| **Number of Observations Used** | 224 |

| **Response Profile** | | |
| --- | --- | --- |
| **Ordered Value** | **late_ab** | **Total Frequency** |
| **1** | 0 | 84 |
| **2** | 1 | 140 |

| ***Probability modeled is late_ab=1.*** |
| --- |

| ***Forward Selection Procedure*** |
| --- |

| ***Step 0. Intercept entered:*** |
| --- |

| **Model Convergence Status** |
| --- |
| Convergence criterion (GCONV=1E-8) satisfied. |

| **-2 Log L** | = | 296.380 |
| --- | --- | --- |

| **Analysis of Maximum Likelihood Estimates** | | | | | |
| --- | --- | --- | --- | --- | --- |
| **Parameter** | **DF** | **Estimate** | **Standard Error** | **Wald Chi-Square** | **Pr > ChiSq** |
| **Intercept** | 1 | 0.5108 | 0.1380 | 13.6995 | 0.0002 |

| **Residual Chi-Square Test** | | |
| --- | --- | --- |
| **Chi-Square** | **DF** | **Pr > ChiSq** |
| 70.8880 | 34 | 0.0002 |

| **Analysis of Effects Eligible for Entry** | | | |
| --- | --- | --- | --- |
| **Effect** | **DF** | **Score Chi-Square** | **Pr > ChiSq** |
| **age** | 1 | 9.1550 | 0.0025 |
| **Male** | 1 | 2.1247 | 0.1449 |
| **admission_type** | 1 | 0.4762 | 0.4902 |
| **apache_score** | 1 | 7.3942 | 0.0065 |
| **sofa** | 1 | 3.0136 | 0.0826 |
| **corticosteroids** | 1 | 2.8187 | 0.0932 |
| **Respiratory_infectio** | 1 | 8.8076 | 0.0030 |
| **Intrabdominal_infect** | 1 | 3.5265 | 0.0604 |
| **Urinary_tract_infect** | 1 | 0.1954 | 0.6585 |
| **Skin_soft_tissue_inf** | 1 | 2.1894 | 0.1390 |
| **Cardiovascular** | 1 | 3.3600 | 0.0668 |
| **Non_hematologic_mali** | 1 | 0.1299 | 0.7186 |
| **Chronic_Kidney_Disea** | 1 | 3.9570 | 0.0467 |
| **comor3** | 1 | 1.4341 | 0.2311 |
| **Hematologic_malignan** | 1 | 0.1929 | 0.6605 |
| **Autoimmune_diseases** | 1 | 0.2458 | 0.6201 |
| **Solid_organ_transpla** | 1 | 1.1417 | 0.2853 |
| **comor12** | 1 | 0.2715 | 0.6023 |
| **any_use_of_nephrtoxi** | 1 | 0.0750 | 0.7841 |
| **vasopressors** | 1 | 2.9661 | 0.0850 |
| **procalcitonin_upon_a** | 1 | 0.9592 | 0.3274 |
| **cultures** | 1 | 1.5937 | 0.2068 |
| **baseline_creatinin** | 1 | 4.1631 | 0.0413 |
| **Upon_enrollment_SrCr** | 1 | 14.4595 | 0.0001 |
| **Srcr_24_hour_post_ad** | 1 | 13.1315 | 0.0003 |
| **SrCr_48_hour_post_ad** | 1 | 14.9209 | 0.0001 |
| **acute_kidney_injury_** | 1 | 3.9827 | 0.0460 |
| **Vancomycin** | 1 | 0.4762 | 0.4902 |
| **lactic_acid_updated** | 1 | 2.7045 | 0.1001 |
| **Aminoglycosides** | 1 | 1.2108 | 0.2712 |
| **other_nephrotoxic_dr** | 1 | 6.7879 | 0.0092 |
| **beta_lactam_choice** | 1 | 3.1387 | 0.0765 |
| **escalation_of_therap** | 1 | 0.5194 | 0.4711 |
| **institute** | 1 | 1.0382 | 0.3082 |

| ***Step 1. Effect SrCr_48_hour_post_ad entered:*** |
| --- |

| **Model Convergence Status** |
| --- |
| Convergence criterion (GCONV=1E-8) satisfied. |

| **Model Fit Statistics** | | |
| --- | --- | --- |
| **Criterion** | **Intercept Only** | **Intercept and Covariates** |
| **AIC** | 298.380 | 285.354 |
| **SC** | 301.792 | 292.177 |
| **-2 Log L** | 296.380 | 281.354 |

| **Testing Global Null Hypothesis: BETA=0** | | | |
| --- | --- | --- | --- |
| **Test** | **Chi-Square** | **DF** | **Pr > ChiSq** |
| **Likelihood Ratio** | 15.0264 | 1 | 0.0001 |
| **Score** | 14.9209 | 1 | 0.0001 |
| **Wald** | 12.8191 | 1 | 0.0003 |

| **Analysis of Maximum Likelihood Estimates** | | | | | |
| --- | --- | --- | --- | --- | --- |
| **Parameter** | **DF** | **Estimate** | **Standard Error** | **Wald Chi-Square** | **Pr > ChiSq** |
| **Intercept** | 1 | 1.3345 | 0.2696 | 24.5112 | <.0001 |
| **SrCr_48_hour_post_ad** | 1 | -0.00432 | 0.00121 | 12.8191 | 0.0003 |

| **Odds Ratio Estimates** | | | |
| --- | --- | --- | --- |
| **Effect** | **Point Estimate** | **95% Wald Confidence Limits** | |
| **SrCr_48_hour_post_ad** | 0.996 | 0.993 | 0.998 |

| **Association of Predicted Probabilities and Observed Responses** | | | |
| --- | --- | --- | --- |
| **Percent Concordant** | 64.1 | **Somers' D** | 0.286 |
| **Percent Discordant** | 35.5 | **Gamma** | 0.287 |
| **Percent Tied** | 0.3 | **Tau-a** | 0.135 |
| **Pairs** | 11760 | **c** | 0.643 |

| **Residual Chi-Square Test** | | |
| --- | --- | --- |
| **Chi-Square** | **DF** | **Pr > ChiSq** |
| 59.4021 | 33 | 0.0032 |

| **Analysis of Effects Eligible for Entry** | | | |
| --- | --- | --- | --- |
| **Effect** | **DF** | **Score Chi-Square** | **Pr > ChiSq** |
| **age** | 1 | 9.3904 | 0.0022 |
| **Male** | 1 | 3.9627 | 0.0465 |
| **admission_type** | 1 | 0.5795 | 0.4465 |
| **apache_score** | 1 | 3.9937 | 0.0457 |
| **sofa** | 1 | 1.1237 | 0.2891 |
| **corticosteroids** | 1 | 3.9716 | 0.0463 |
| **Respiratory_infectio** | 1 | 9.8245 | 0.0017 |
| **Intrabdominal_infect** | 1 | 3.6264 | 0.0569 |
| **Urinary_tract_infect** | 1 | 0.9864 | 0.3206 |
| **Skin_soft_tissue_inf** | 1 | 2.6055 | 0.1065 |
| **Cardiovascular** | 1 | 2.3025 | 0.1292 |
| **Non_hematologic_mali** | 1 | 0.2349 | 0.6279 |
| **Chronic_Kidney_Disea** | 1 | 1.3021 | 0.2538 |
| **comor3** | 1 | 1.1356 | 0.2866 |
| **Hematologic_malignan** | 1 | 0.6297 | 0.4274 |
| **Autoimmune_diseases** | 1 | 0.1434 | 0.7049 |
| **Solid_organ_transpla** | 1 | 1.0820 | 0.2982 |
| **comor12** | 1 | 0.0577 | 0.8101 |
| **any_use_of_nephrtoxi** | 1 | 0.0085 | 0.9266 |
| **vasopressors** | 1 | 1.6650 | 0.1969 |
| **procalcitonin_upon_a** | 1 | 0.2310 | 0.6308 |
| **cultures** | 1 | 3.1139 | 0.0776 |
| **baseline_creatinin** | 1 | 0.3823 | 0.5364 |
| **Upon_enrollment_SrCr** | 1 | 3.5769 | 0.0586 |
| **Srcr_24_hour_post_ad** | 1 | 0.2435 | 0.6217 |
| **acute_kidney_injury_** | 1 | 0.6241 | 0.4295 |
| **Vancomycin** | 1 | 0.3193 | 0.5720 |
| **lactic_acid_updated** | 1 | 1.7277 | 0.1887 |
| **Aminoglycosides** | 1 | 0.9563 | 0.3281 |
| **other_nephrotoxic_dr** | 1 | 7.4273 | 0.0064 |
| **beta_lactam_choice** | 1 | 3.1168 | 0.0775 |
| **escalation_of_therap** | 1 | 0.8915 | 0.3451 |
| **institute** | 1 | 1.1535 | 0.2828 |

| ***Step 2. Effect Respiratory_infectio entered:*** |
| --- |

| **Model Convergence Status** |
| --- |
| Convergence criterion (GCONV=1E-8) satisfied. |

| **Model Fit Statistics** | | |
| --- | --- | --- |
| **Criterion** | **Intercept Only** | **Intercept and Covariates** |
| **AIC** | 298.380 | 277.415 |
| **SC** | 301.792 | 287.650 |
| **-2 Log L** | 296.380 | 271.415 |

| **Testing Global Null Hypothesis: BETA=0** | | | |
| --- | --- | --- | --- |
| **Test** | **Chi-Square** | **DF** | **Pr > ChiSq** |
| **Likelihood Ratio** | 24.9654 | 2 | <.0001 |
| **Score** | 23.9835 | 2 | <.0001 |
| **Wald** | 20.6973 | 2 | <.0001 |

| **Analysis of Maximum Likelihood Estimates** | | | | | |
| --- | --- | --- | --- | --- | --- |
| **Parameter** | **DF** | **Estimate** | **Standard Error** | **Wald Chi-Square** | **Pr > ChiSq** |
| **Intercept** | 1 | 0.9478 | 0.2947 | 10.3426 | 0.0013 |
| **Respiratory_infectio** | 1 | 0.9172 | 0.2962 | 9.5915 | 0.0020 |
| **SrCr_48_hour_post_ad** | 1 | -0.00460 | 0.00124 | 13.7035 | 0.0002 |

| **Odds Ratio Estimates** | | | |
| --- | --- | --- | --- |
| **Effect** | **Point Estimate** | **95% Wald Confidence Limits** | |
| **Respiratory_infectio** | 2.502 | 1.400 | 4.471 |
| **SrCr_48_hour_post_ad** | 0.995 | 0.993 | 0.998 |

| **Association of Predicted Probabilities and Observed Responses** | | | |
| --- | --- | --- | --- |
| **Percent Concordant** | 67.4 | **Somers' D** | 0.351 |
| **Percent Discordant** | 32.3 | **Gamma** | 0.352 |
| **Percent Tied** | 0.2 | **Tau-a** | 0.165 |
| **Pairs** | 11760 | **c** | 0.676 |

| **Residual Chi-Square Test** | | |
| --- | --- | --- |
| **Chi-Square** | **DF** | **Pr > ChiSq** |
| 50.6087 | 32 | 0.0195 |

| **Analysis of Effects Eligible for Entry** | | | |
| --- | --- | --- | --- |
| **Effect** | **DF** | **Score Chi-Square** | **Pr > ChiSq** |
| **age** | 1 | 11.7260 | 0.0006 |
| **Male** | 1 | 4.0630 | 0.0438 |
| **admission_type** | 1 | 0.0551 | 0.8143 |
| **apache_score** | 1 | 7.1838 | 0.0074 |
| **sofa** | 1 | 1.9751 | 0.1599 |
| **corticosteroids** | 1 | 4.5496 | 0.0329 |
| **Intrabdominal_infect** | 1 | 0.4257 | 0.5141 |
| **Urinary_tract_infect** | 1 | 0.0021 | 0.9632 |
| **Skin_soft_tissue_inf** | 1 | 0.7832 | 0.3762 |
| **Cardiovascular** | 1 | 3.6091 | 0.0575 |
| **Non_hematologic_mali** | 1 | 0.0066 | 0.9351 |
| **Chronic_Kidney_Disea** | 1 | 1.1184 | 0.2903 |
| **comor3** | 1 | 1.5997 | 0.2059 |
| **Hematologic_malignan** | 1 | 0.1618 | 0.6875 |
| **Autoimmune_diseases** | 1 | 0.3706 | 0.5427 |
| **Solid_organ_transpla** | 1 | 0.5232 | 0.4695 |
| **comor12** | 1 | 0.2045 | 0.6511 |
| **any_use_of_nephrtoxi** | 1 | 0.0772 | 0.7812 |
| **vasopressors** | 1 | 1.5949 | 0.2066 |
| **procalcitonin_upon_a** | 1 | 0.0169 | 0.8965 |
| **cultures** | 1 | 1.7538 | 0.1854 |
| **baseline_creatinin** | 1 | 0.7598 | 0.3834 |
| **Upon_enrollment_SrCr** | 1 | 2.5294 | 0.1117 |
| **Srcr_24_hour_post_ad** | 1 | 0.1388 | 0.7095 |
| **acute_kidney_injury_** | 1 | 0.1650 | 0.6846 |
| **Vancomycin** | 1 | 0.7926 | 0.3733 |
| **lactic_acid_updated** | 1 | 0.8870 | 0.3463 |
| **Aminoglycosides** | 1 | 1.4645 | 0.2262 |
| **other_nephrotoxic_dr** | 1 | 7.5435 | 0.0060 |
| **beta_lactam_choice** | 1 | 2.2827 | 0.1308 |
| **escalation_of_therap** | 1 | 0.7199 | 0.3962 |
| **institute** | 1 | 0.8752 | 0.3495 |

| ***Step 3. Effect age entered:*** |
| --- |

| **Model Convergence Status** |
| --- |
| Convergence criterion (GCONV=1E-8) satisfied. |

| **Model Fit Statistics** | | |
| --- | --- | --- |
| **Criterion** | **Intercept Only** | **Intercept and Covariates** |
| **AIC** | 298.380 | 267.128 |
| **SC** | 301.792 | 280.775 |
| **-2 Log L** | 296.380 | 259.128 |

| **Testing Global Null Hypothesis: BETA=0** | | | |
| --- | --- | --- | --- |
| **Test** | **Chi-Square** | **DF** | **Pr > ChiSq** |
| **Likelihood Ratio** | 37.2521 | 3 | <.0001 |
| **Score** | 34.3600 | 3 | <.0001 |
| **Wald** | 28.7011 | 3 | <.0001 |

| **Analysis of Maximum Likelihood Estimates** | | | | | |
| --- | --- | --- | --- | --- | --- |
| **Parameter** | **DF** | **Estimate** | **Standard Error** | **Wald Chi-Square** | **Pr > ChiSq** |
| **Intercept** | 1 | 3.0428 | 0.7102 | 18.3562 | <.0001 |
| **age** | 1 | -0.0333 | 0.00997 | 11.1694 | 0.0008 |
| **Respiratory_infectio** | 1 | 1.0704 | 0.3112 | 11.8348 | 0.0006 |
| **SrCr_48_hour_post_ad** | 1 | -0.00480 | 0.00128 | 14.1378 | 0.0002 |

| **Odds Ratio Estimates** | | | |
| --- | --- | --- | --- |
| **Effect** | **Point Estimate** | **95% Wald Confidence Limits** | |
| **age** | 0.967 | 0.948 | 0.986 |
| **Respiratory_infectio** | 2.917 | 1.585 | 5.367 |
| **SrCr_48_hour_post_ad** | 0.995 | 0.993 | 0.998 |

| **Association of Predicted Probabilities and Observed Responses** | | | |
| --- | --- | --- | --- |
| **Percent Concordant** | 73.1 | **Somers' D** | 0.463 |
| **Percent Discordant** | 26.9 | **Gamma** | 0.463 |
| **Percent Tied** | 0.0 | **Tau-a** | 0.218 |
| **Pairs** | 11760 | **c** | 0.731 |

| **Residual Chi-Square Test** | | |
| --- | --- | --- |
| **Chi-Square** | **DF** | **Pr > ChiSq** |
| 40.9367 | 31 | 0.1092 |

| **Analysis of Effects Eligible for Entry** | | | |
| --- | --- | --- | --- |
| **Effect** | **DF** | **Score Chi-Square** | **Pr > ChiSq** |
| **Male** | 1 | 1.9479 | 0.1628 |
| **admission_type** | 1 | 0.0388 | 0.8438 |
| **apache_score** | 1 | 3.5295 | 0.0603 |
| **sofa** | 1 | 1.3485 | 0.2455 |
| **corticosteroids** | 1 | 5.9162 | 0.0150 |
| **Intrabdominal_infect** | 1 | 0.5048 | 0.4774 |
| **Urinary_tract_infect** | 1 | 0.0269 | 0.8698 |
| **Skin_soft_tissue_inf** | 1 | 1.1703 | 0.2793 |
| **Cardiovascular** | 1 | 0.0377 | 0.8460 |
| **Non_hematologic_mali** | 1 | 0.0906 | 0.7634 |
| **Chronic_Kidney_Disea** | 1 | 0.3409 | 0.5593 |
| **comor3** | 1 | 2.2340 | 0.1350 |
| **Hematologic_malignan** | 1 | 0.4795 | 0.4887 |
| **Autoimmune_diseases** | 1 | 0.0101 | 0.9199 |
| **Solid_organ_transpla** | 1 | 0.6060 | 0.4363 |
| **comor12** | 1 | 0.0439 | 0.8340 |
| **any_use_of_nephrtoxi** | 1 | 0.2650 | 0.6067 |
| **vasopressors** | 1 | 1.1236 | 0.2892 |
| **procalcitonin_upon_a** | 1 | 0.2904 | 0.5899 |
| **cultures** | 1 | 0.3980 | 0.5281 |
| **baseline_creatinin** | 1 | 0.1531 | 0.6956 |
| **Upon_enrollment_SrCr** | 1 | 2.8081 | 0.0938 |
| **Srcr_24_hour_post_ad** | 1 | 0.2266 | 0.6341 |
| **acute_kidney_injury_** | 1 | 0.5134 | 0.4737 |
| **Vancomycin** | 1 | 1.5034 | 0.2201 |
| **lactic_acid_updated** | 1 | 0.9908 | 0.3195 |
| **Aminoglycosides** | 1 | 1.3401 | 0.2470 |
| **other_nephrotoxic_dr** | 1 | 6.3117 | 0.0120 |
| **beta_lactam_choice** | 1 | 1.9957 | 0.1577 |
| **escalation_of_therap** | 1 | 1.5309 | 0.2160 |
| **institute** | 1 | 0.3341 | 0.5633 |

| ***Step 4. Effect other_nephrotoxic_dr entered:*** |
| --- |

| **Model Convergence Status** |
| --- |
| Quasi-complete separation of data points detected. |

| **Warning:** | The maximum likelihood estimate may not exist. |  |
| --- | --- | --- |
| **Warning:** | The LOGISTIC procedure continues in spite of the above warning. Results shown are based on the last maximum likelihood iteration. Validity of the model fit is questionable. | |

| **Model Fit Statistics** | | |
| --- | --- | --- |
| **Criterion** | **Intercept Only** | **Intercept and Covariates** |
| **AIC** | 298.380 | 261.503 |
| **SC** | 301.792 | 278.561 |
| **-2 Log L** | 296.380 | 251.503 |

| **Testing Global Null Hypothesis: BETA=0** | | | |
| --- | --- | --- | --- |
| **Test** | **Chi-Square** | **DF** | **Pr > ChiSq** |
| **Likelihood Ratio** | 44.8774 | 4 | <.0001 |
| **Score** | 40.4166 | 4 | <.0001 |
| **Wald** | 28.4030 | 4 | <.0001 |

| **Analysis of Maximum Likelihood Estimates** | | | | | |
| --- | --- | --- | --- | --- | --- |
| **Parameter** | **DF** | **Estimate** | **Standard Error** | **Wald Chi-Square** | **Pr > ChiSq** |
| **Intercept** | 1 | 3.0437 | 0.7172 | 18.0115 | <.0001 |
| **age** | 1 | -0.0324 | 0.0101 | 10.3546 | 0.0013 |
| **Respiratory_infectio** | 1 | 1.0856 | 0.3164 | 11.7745 | 0.0006 |
| **SrCr_48_hour_post_ad** | 1 | -0.00487 | 0.00128 | 14.4005 | 0.0001 |
| **other_nephrotoxic_dr** | 1 | -14.8363 | 638.6 | 0.0005 | 0.9815 |

| **Odds Ratio Estimates** | | | |
| --- | --- | --- | --- |
| **Effect** | **Point Estimate** | **95% Wald Confidence Limits** | |
| **age** | 0.968 | 0.949 | 0.987 |
| **Respiratory_infectio** | 2.961 | 1.593 | 5.505 |
| **SrCr_48_hour_post_ad** | 0.995 | 0.993 | 0.998 |
| **other_nephrotoxic_dr** | <0.001 | <0.001 | >999.999 |

| **Association of Predicted Probabilities and Observed Responses** | | | |
| --- | --- | --- | --- |
| **Percent Concordant** | 74.5 | **Somers' D** | 0.491 |
| **Percent Discordant** | 25.5 | **Gamma** | 0.491 |
| **Percent Tied** | 0.0 | **Tau-a** | 0.231 |
| **Pairs** | 11760 | **c** | 0.745 |

| **Residual Chi-Square Test** | | |
| --- | --- | --- |
| **Chi-Square** | **DF** | **Pr > ChiSq** |
| 36.8478 | 30 | 0.1816 |

| **Analysis of Effects Eligible for Entry** | | | |
| --- | --- | --- | --- |
| **Effect** | **DF** | **Score Chi-Square** | **Pr > ChiSq** |
| **Male** | 1 | 1.3633 | 0.2430 |
| **admission_type** | 1 | 0.0317 | 0.8587 |
| **apache_score** | 1 | 3.6675 | 0.0555 |
| **sofa** | 1 | 1.5745 | 0.2095 |
| **corticosteroids** | 1 | 5.6911 | 0.0171 |
| **Intrabdominal_infect** | 1 | 0.8302 | 0.3622 |
| **Urinary_tract_infect** | 1 | 0.1951 | 0.6587 |
| **Skin_soft_tissue_inf** | 1 | 1.4298 | 0.2318 |
| **Cardiovascular** | 1 | 0.0779 | 0.7802 |
| **Non_hematologic_mali** | 1 | 0.0496 | 0.8237 |
| **Chronic_Kidney_Disea** | 1 | 0.1587 | 0.6903 |
| **comor3** | 1 | 2.5094 | 0.1132 |
| **Hematologic_malignan** | 1 | 0.5787 | 0.4468 |
| **Autoimmune_diseases** | 1 | 0.0028 | 0.9579 |
| **Solid_organ_transpla** | 1 | 2.0861 | 0.1486 |
| **comor12** | 1 | 0.0710 | 0.7899 |
| **any_use_of_nephrtoxi** | 1 | 0.4067 | 0.5236 |
| **vasopressors** | 1 | 0.8600 | 0.3538 |
| **procalcitonin_upon_a** | 1 | 0.3833 | 0.5358 |
| **cultures** | 1 | 0.1234 | 0.7253 |
| **baseline_creatinin** | 1 | 0.1877 | 0.6648 |
| **Upon_enrollment_SrCr** | 1 | 3.1106 | 0.0778 |
| **Srcr_24_hour_post_ad** | 1 | 0.1490 | 0.6995 |
| **acute_kidney_injury_** | 1 | 0.3013 | 0.5831 |
| **Vancomycin** | 1 | 0.5943 | 0.4407 |
| **lactic_acid_updated** | 1 | 0.7682 | 0.3808 |
| **Aminoglycosides** | 1 | 1.2845 | 0.2571 |
| **beta_lactam_choice** | 1 | 1.2388 | 0.2657 |
| **escalation_of_therap** | 1 | 1.7940 | 0.1804 |
| **institute** | 1 | 0.3193 | 0.5720 |

| ***Step 5. Effect corticosteroids entered:*** |
| --- |

| **Model Convergence Status** |
| --- |
| Quasi-complete separation of data points detected. |

| **Warning:** | The maximum likelihood estimate may not exist. |  |
| --- | --- | --- |
| **Warning:** | The LOGISTIC procedure continues in spite of the above warning. Results shown are based on the last maximum likelihood iteration. Validity of the model fit is questionable. | |

| **Model Fit Statistics** | | |
| --- | --- | --- |
| **Criterion** | **Intercept Only** | **Intercept and Covariates** |
| **AIC** | 298.380 | 257.823 |
| **SC** | 301.792 | 278.293 |
| **-2 Log L** | 296.380 | 245.823 |

| **Testing Global Null Hypothesis: BETA=0** | | | |
| --- | --- | --- | --- |
| **Test** | **Chi-Square** | **DF** | **Pr > ChiSq** |
| **Likelihood Ratio** | 50.5576 | 5 | <.0001 |
| **Score** | 45.5601 | 5 | <.0001 |
| **Wald** | 32.4676 | 5 | <.0001 |

| **Analysis of Maximum Likelihood Estimates** | | | | | |
| --- | --- | --- | --- | --- | --- |
| **Parameter** | **DF** | **Estimate** | **Standard Error** | **Wald Chi-Square** | **Pr > ChiSq** |
| **Intercept** | 1 | 2.7232 | 0.7283 | 13.9797 | 0.0002 |
| **age** | 1 | -0.0346 | 0.0102 | 11.4905 | 0.0007 |
| **corticosteroids** | 1 | 0.7670 | 0.3246 | 5.5820 | 0.0181 |
| **Respiratory_infectio** | 1 | 1.1303 | 0.3208 | 12.4168 | 0.0004 |
| **SrCr_48_hour_post_ad** | 1 | -0.00509 | 0.00129 | 15.4645 | <.0001 |
| **other_nephrotoxic_dr** | 1 | -14.8006 | 621.7 | 0.0006 | 0.9810 |

| **Odds Ratio Estimates** | | | |
| --- | --- | --- | --- |
| **Effect** | **Point Estimate** | **95% Wald Confidence Limits** | |
| **age** | 0.966 | 0.947 | 0.986 |
| **corticosteroids** | 2.153 | 1.140 | 4.069 |
| **Respiratory_infectio** | 3.097 | 1.651 | 5.807 |
| **SrCr_48_hour_post_ad** | 0.995 | 0.992 | 0.997 |
| **other_nephrotoxic_dr** | <0.001 | <0.001 | >999.999 |

| **Association of Predicted Probabilities and Observed Responses** | | | |
| --- | --- | --- | --- |
| **Percent Concordant** | 75.9 | **Somers' D** | 0.517 |
| **Percent Discordant** | 24.1 | **Gamma** | 0.517 |
| **Percent Tied** | 0.0 | **Tau-a** | 0.244 |
| **Pairs** | 11760 | **c** | 0.759 |

| **Residual Chi-Square Test** | | |
| --- | --- | --- |
| **Chi-Square** | **DF** | **Pr > ChiSq** |
| 31.6984 | 29 | 0.3333 |

| **Analysis of Effects Eligible for Entry** | | | |
| --- | --- | --- | --- |
| **Effect** | **DF** | **Score Chi-Square** | **Pr > ChiSq** |
| **Male** | 1 | 1.1944 | 0.2744 |
| **admission_type** | 1 | 0.0336 | 0.8545 |
| **apache_score** | 1 | 4.3331 | 0.0374 |
| **sofa** | 1 | 3.3956 | 0.0654 |
| **Intrabdominal_infect** | 1 | 0.7107 | 0.3992 |
| **Urinary_tract_infect** | 1 | 0.4667 | 0.4945 |
| **Skin_soft_tissue_inf** | 1 | 1.3850 | 0.2392 |
| **Cardiovascular** | 1 | 0.0025 | 0.9598 |
| **Non_hematologic_mali** | 1 | 0.0895 | 0.7648 |
| **Chronic_Kidney_Disea** | 1 | 0.0482 | 0.8262 |
| **comor3** | 1 | 2.7115 | 0.0996 |
| **Hematologic_malignan** | 1 | 1.0740 | 0.3000 |
| **Autoimmune_diseases** | 1 | 0.0092 | 0.9236 |
| **Solid_organ_transpla** | 1 | 1.5905 | 0.2073 |
| **comor12** | 1 | 0.0471 | 0.8282 |
| **any_use_of_nephrtoxi** | 1 | 0.0555 | 0.8138 |
| **vasopressors** | 1 | 4.8817 | 0.0271 |
| **procalcitonin_upon_a** | 1 | 0.0851 | 0.7706 |
| **cultures** | 1 | 0.2221 | 0.6374 |
| **baseline_creatinin** | 1 | 0.0509 | 0.8215 |
| **Upon_enrollment_SrCr** | 1 | 4.1966 | 0.0405 |
| **Srcr_24_hour_post_ad** | 1 | 0.4012 | 0.5265 |
| **acute_kidney_injury_** | 1 | 1.0667 | 0.3017 |
| **Vancomycin** | 1 | 0.0051 | 0.9430 |
| **lactic_acid_updated** | 1 | 1.7618 | 0.1844 |
| **Aminoglycosides** | 1 | 1.0159 | 0.3135 |
| **beta_lactam_choice** | 1 | 1.3112 | 0.2522 |
| **escalation_of_therap** | 1 | 0.9950 | 0.3185 |
| **institute** | 1 | 0.7498 | 0.3865 |

| ***Step 6. Effect vasopressors entered:*** |
| --- |

| **Model Convergence Status** |
| --- |
| Quasi-complete separation of data points detected. |

| **Warning:** | The maximum likelihood estimate may not exist. |  |
| --- | --- | --- |
| **Warning:** | The LOGISTIC procedure continues in spite of the above warning. Results shown are based on the last maximum likelihood iteration. Validity of the model fit is questionable. | |

| **Model Fit Statistics** | | |
| --- | --- | --- |
| **Criterion** | **Intercept Only** | **Intercept and Covariates** |
| **AIC** | 298.380 | 254.713 |
| **SC** | 301.792 | 278.595 |
| **-2 Log L** | 296.380 | 240.713 |

| **Testing Global Null Hypothesis: BETA=0** | | | |
| --- | --- | --- | --- |
| **Test** | **Chi-Square** | **DF** | **Pr > ChiSq** |
| **Likelihood Ratio** | 55.6673 | 6 | <.0001 |
| **Score** | 49.4856 | 6 | <.0001 |
| **Wald** | 34.7726 | 6 | <.0001 |

| **Analysis of Maximum Likelihood Estimates** | | | | | |
| --- | --- | --- | --- | --- | --- |
| **Parameter** | **DF** | **Estimate** | **Standard Error** | **Wald Chi-Square** | **Pr > ChiSq** |
| **Intercept** | 1 | 3.4993 | 0.8357 | 17.5351 | <.0001 |
| **age** | 1 | -0.0349 | 0.0103 | 11.4650 | 0.0007 |
| **corticosteroids** | 1 | 1.1452 | 0.3723 | 9.4640 | 0.0021 |
| **Respiratory_infectio** | 1 | 1.1606 | 0.3260 | 12.6752 | 0.0004 |
| **vasopressors** | 1 | -1.2003 | 0.5531 | 4.7104 | 0.0300 |
| **SrCr_48_hour_post_ad** | 1 | -0.00490 | 0.00131 | 14.0801 | 0.0002 |
| **other_nephrotoxic_dr** | 1 | -14.6700 | 608.9 | 0.0006 | 0.9808 |

| **Odds Ratio Estimates** | | | |
| --- | --- | --- | --- |
| **Effect** | **Point Estimate** | **95% Wald Confidence Limits** | |
| **age** | 0.966 | 0.946 | 0.985 |
| **corticosteroids** | 3.143 | 1.515 | 6.519 |
| **Respiratory_infectio** | 3.192 | 1.685 | 6.047 |
| **vasopressors** | 0.301 | 0.102 | 0.890 |
| **SrCr_48_hour_post_ad** | 0.995 | 0.993 | 0.998 |
| **other_nephrotoxic_dr** | <0.001 | <0.001 | >999.999 |

| **Association of Predicted Probabilities and Observed Responses** | | | |
| --- | --- | --- | --- |
| **Percent Concordant** | 77.3 | **Somers' D** | 0.547 |
| **Percent Discordant** | 22.7 | **Gamma** | 0.547 |
| **Percent Tied** | 0.0 | **Tau-a** | 0.258 |
| **Pairs** | 11760 | **c** | 0.773 |

| **Residual Chi-Square Test** | | |
| --- | --- | --- |
| **Chi-Square** | **DF** | **Pr > ChiSq** |
| 27.1773 | 28 | 0.5086 |

| **Analysis of Effects Eligible for Entry** | | | |
| --- | --- | --- | --- |
| **Effect** | **DF** | **Score Chi-Square** | **Pr > ChiSq** |
| **Male** | 1 | 1.2667 | 0.2604 |
| **admission_type** | 1 | 0.1221 | 0.7268 |
| **apache_score** | 1 | 3.3503 | 0.0672 |
| **sofa** | 1 | 1.4583 | 0.2272 |
| **Intrabdominal_infect** | 1 | 0.4725 | 0.4919 |
| **Urinary_tract_infect** | 1 | 0.1706 | 0.6796 |
| **Skin_soft_tissue_inf** | 1 | 0.8958 | 0.3439 |
| **Cardiovascular** | 1 | 0.0000 | 0.9965 |
| **Non_hematologic_mali** | 1 | 0.0034 | 0.9536 |
| **Chronic_Kidney_Disea** | 1 | 0.1923 | 0.6610 |
| **comor3** | 1 | 3.0029 | 0.0831 |
| **Hematologic_malignan** | 1 | 1.8123 | 0.1782 |
| **Autoimmune_diseases** | 1 | 0.0087 | 0.9257 |
| **Solid_organ_transpla** | 1 | 1.6160 | 0.2037 |
| **comor12** | 1 | 0.0079 | 0.9293 |
| **any_use_of_nephrtoxi** | 1 | 0.2731 | 0.6013 |
| **procalcitonin_upon_a** | 1 | 0.0564 | 0.8123 |
| **cultures** | 1 | 0.1537 | 0.6951 |
| **baseline_creatinin** | 1 | 0.4331 | 0.5105 |
| **Upon_enrollment_SrCr** | 1 | 5.1120 | 0.0238 |
| **Srcr_24_hour_post_ad** | 1 | 0.7980 | 0.3717 |
| **acute_kidney_injury_** | 1 | 0.5947 | 0.4406 |
| **Vancomycin** | 1 | 0.0431 | 0.8356 |
| **lactic_acid_updated** | 1 | 1.1550 | 0.2825 |
| **Aminoglycosides** | 1 | 1.0415 | 0.3075 |
| **beta_lactam_choice** | 1 | 1.0394 | 0.3080 |
| **escalation_of_therap** | 1 | 1.3846 | 0.2393 |
| **institute** | 1 | 1.0479 | 0.3060 |

| ***Step 7. Effect Upon_enrollment_SrCr entered:*** |
| --- |

| **Model Convergence Status** |
| --- |
| Quasi-complete separation of data points detected. |

| **Warning:** | The maximum likelihood estimate may not exist. |  |
| --- | --- | --- |
| **Warning:** | The LOGISTIC procedure continues in spite of the above warning. Results shown are based on the last maximum likelihood iteration. Validity of the model fit is questionable. | |

| **Model Fit Statistics** | | |
| --- | --- | --- |
| **Criterion** | **Intercept Only** | **Intercept and Covariates** |
| **AIC** | 298.380 | 251.352 |
| **SC** | 301.792 | 278.645 |
| **-2 Log L** | 296.380 | 235.352 |

| **Testing Global Null Hypothesis: BETA=0** | | | |
| --- | --- | --- | --- |
| **Test** | **Chi-Square** | **DF** | **Pr > ChiSq** |
| **Likelihood Ratio** | 61.0284 | 7 | <.0001 |
| **Score** | 53.1843 | 7 | <.0001 |
| **Wald** | 36.1153 | 7 | <.0001 |

| **Analysis of Maximum Likelihood Estimates** | | | | | |
| --- | --- | --- | --- | --- | --- |
| **Parameter** | **DF** | **Estimate** | **Standard Error** | **Wald Chi-Square** | **Pr > ChiSq** |
| **Intercept** | 1 | 4.1352 | 0.9094 | 20.6776 | <.0001 |
| **age** | 1 | -0.0363 | 0.0105 | 11.9167 | 0.0006 |
| **corticosteroids** | 1 | 1.2861 | 0.3826 | 11.2981 | 0.0008 |
| **Respiratory_infectio** | 1 | 1.1323 | 0.3301 | 11.7694 | 0.0006 |
| **vasopressors** | 1 | -1.3249 | 0.5599 | 5.5995 | 0.0180 |
| **Upon_enrollment_SrCr** | 1 | -0.00450 | 0.00203 | 4.9216 | 0.0265 |
| **SrCr_48_hour_post_ad** | 1 | -0.00265 | 0.00163 | 2.6365 | 0.1044 |
| **other_nephrotoxic_dr** | 1 | -14.8439 | 590.3 | 0.0006 | 0.9799 |

| **Odds Ratio Estimates** | | | |
| --- | --- | --- | --- |
| **Effect** | **Point Estimate** | **95% Wald Confidence Limits** | |
| **age** | 0.964 | 0.945 | 0.984 |
| **corticosteroids** | 3.619 | 1.709 | 7.660 |
| **Respiratory_infectio** | 3.103 | 1.625 | 5.925 |
| **vasopressors** | 0.266 | 0.089 | 0.797 |
| **Upon_enrollment_SrCr** | 0.996 | 0.992 | 0.999 |
| **SrCr_48_hour_post_ad** | 0.997 | 0.994 | 1.001 |
| **other_nephrotoxic_dr** | <0.001 | <0.001 | >999.999 |

| **Association of Predicted Probabilities and Observed Responses** | | | |
| --- | --- | --- | --- |
| **Percent Concordant** | 78.9 | **Somers' D** | 0.578 |
| **Percent Discordant** | 21.1 | **Gamma** | 0.578 |
| **Percent Tied** | 0.0 | **Tau-a** | 0.272 |
| **Pairs** | 11760 | **c** | 0.789 |

| **Residual Chi-Square Test** | | |
| --- | --- | --- |
| **Chi-Square** | **DF** | **Pr > ChiSq** |
| 22.6952 | 27 | 0.7013 |

| **Analysis of Effects Eligible for Entry** | | | |
| --- | --- | --- | --- |
| **Effect** | **DF** | **Score Chi-Square** | **Pr > ChiSq** |
| **Male** | 1 | 1.0724 | 0.3004 |
| **admission_type** | 1 | 0.0067 | 0.9349 |
| **apache_score** | 1 | 1.6828 | 0.1946 |
| **sofa** | 1 | 1.5908 | 0.2072 |
| **Intrabdominal_infect** | 1 | 0.8861 | 0.3465 |
| **Urinary_tract_infect** | 1 | 0.2544 | 0.6140 |
| **Skin_soft_tissue_inf** | 1 | 1.1872 | 0.2759 |
| **Cardiovascular** | 1 | 0.0161 | 0.8991 |
| **Non_hematologic_mali** | 1 | 0.0833 | 0.7729 |
| **Chronic_Kidney_Disea** | 1 | 0.0043 | 0.9474 |
| **comor3** | 1 | 2.7980 | 0.0944 |
| **Hematologic_malignan** | 1 | 1.5393 | 0.2147 |
| **Autoimmune_diseases** | 1 | 0.0040 | 0.9496 |
| **Solid_organ_transpla** | 1 | 1.4428 | 0.2297 |
| **comor12** | 1 | 0.0161 | 0.8989 |
| **any_use_of_nephrtoxi** | 1 | 0.1009 | 0.7507 |
| **procalcitonin_upon_a** | 1 | 0.0069 | 0.9338 |
| **cultures** | 1 | 0.1809 | 0.6706 |
| **baseline_creatinin** | 1 | 0.0068 | 0.9343 |
| **Srcr_24_hour_post_ad** | 1 | 1.1587 | 0.2817 |
| **acute_kidney_injury_** | 1 | 0.0003 | 0.9862 |
| **Vancomycin** | 1 | 0.0004 | 0.9839 |
| **lactic_acid_updated** | 1 | 1.8319 | 0.1759 |
| **Aminoglycosides** | 1 | 1.4632 | 0.2264 |
| **beta_lactam_choice** | 1 | 1.4023 | 0.2363 |
| **escalation_of_therap** | 1 | 1.6170 | 0.2035 |
| **institute** | 1 | 1.4456 | 0.2292 |

| ***Step 8. Effect comor3 entered:*** |
| --- |

| **Model Convergence Status** |
| --- |
| Quasi-complete separation of data points detected. |

| **Warning:** | The maximum likelihood estimate may not exist. |  |
| --- | --- | --- |
| **Warning:** | The LOGISTIC procedure continues in spite of the above warning. Results shown are based on the last maximum likelihood iteration. Validity of the model fit is questionable. | |

| **Model Fit Statistics** | | |
| --- | --- | --- |
| **Criterion** | **Intercept Only** | **Intercept and Covariates** |
| **AIC** | 298.380 | 250.702 |
| **SC** | 301.792 | 281.407 |
| **-2 Log L** | 296.380 | 232.702 |

| **Testing Global Null Hypothesis: BETA=0** | | | |
| --- | --- | --- | --- |
| **Test** | **Chi-Square** | **DF** | **Pr > ChiSq** |
| **Likelihood Ratio** | 63.6778 | 8 | <.0001 |
| **Score** | 55.4281 | 8 | <.0001 |
| **Wald** | 37.3755 | 8 | <.0001 |

| **Analysis of Maximum Likelihood Estimates** | | | | | |
| --- | --- | --- | --- | --- | --- |
| **Parameter** | **DF** | **Estimate** | **Standard Error** | **Wald Chi-Square** | **Pr > ChiSq** |
| **Intercept** | 1 | 4.2556 | 0.9226 | 21.2774 | <.0001 |
| **age** | 1 | -0.0379 | 0.0107 | 12.5194 | 0.0004 |
| **corticosteroids** | 1 | 1.3143 | 0.3869 | 11.5412 | 0.0007 |
| **Respiratory_infectio** | 1 | 1.1887 | 0.3360 | 12.5159 | 0.0004 |
| **comor3** | 1 | -1.1573 | 0.7118 | 2.6439 | 0.1039 |
| **vasopressors** | 1 | -1.3581 | 0.5659 | 5.7602 | 0.0164 |
| **Upon_enrollment_SrCr** | 1 | -0.00441 | 0.00202 | 4.7640 | 0.0291 |
| **SrCr_48_hour_post_ad** | 1 | -0.00259 | 0.00163 | 2.5391 | 0.1111 |
| **other_nephrotoxic_dr** | 1 | -14.9145 | 587.8 | 0.0006 | 0.9798 |

| **Odds Ratio Estimates** | | | |
| --- | --- | --- | --- |
| **Effect** | **Point Estimate** | **95% Wald Confidence Limits** | |
| **age** | 0.963 | 0.943 | 0.983 |
| **corticosteroids** | 3.722 | 1.744 | 7.945 |
| **Respiratory_infectio** | 3.283 | 1.699 | 6.342 |
| **comor3** | 0.314 | 0.078 | 1.268 |
| **vasopressors** | 0.257 | 0.085 | 0.780 |
| **Upon_enrollment_SrCr** | 0.996 | 0.992 | 1.000 |
| **SrCr_48_hour_post_ad** | 0.997 | 0.994 | 1.001 |
| **other_nephrotoxic_dr** | <0.001 | <0.001 | >999.999 |

| **Association of Predicted Probabilities and Observed Responses** | | | |
| --- | --- | --- | --- |
| **Percent Concordant** | 79.1 | **Somers' D** | 0.583 |
| **Percent Discordant** | 20.9 | **Gamma** | 0.583 |
| **Percent Tied** | 0.0 | **Tau-a** | 0.274 |
| **Pairs** | 11760 | **c** | 0.791 |

| **Residual Chi-Square Test** | | |
| --- | --- | --- |
| **Chi-Square** | **DF** | **Pr > ChiSq** |
| 19.9688 | 26 | 0.7930 |

| **Analysis of Effects Eligible for Entry** | | | |
| --- | --- | --- | --- |
| **Effect** | **DF** | **Score Chi-Square** | **Pr > ChiSq** |
| **Male** | 1 | 1.3112 | 0.2522 |
| **admission_type** | 1 | 0.0447 | 0.8325 |
| **apache_score** | 1 | 1.4714 | 0.2251 |
| **sofa** | 1 | 1.5030 | 0.2202 |
| **Intrabdominal_infect** | 1 | 0.3785 | 0.5384 |
| **Urinary_tract_infect** | 1 | 0.1781 | 0.6730 |
| **Skin_soft_tissue_inf** | 1 | 1.4341 | 0.2311 |
| **Cardiovascular** | 1 | 0.0087 | 0.9255 |
| **Non_hematologic_mali** | 1 | 0.0602 | 0.8062 |
| **Chronic_Kidney_Disea** | 1 | 0.0060 | 0.9381 |
| **Hematologic_malignan** | 1 | 1.2055 | 0.2722 |
| **Autoimmune_diseases** | 1 | 0.0200 | 0.8875 |
| **Solid_organ_transpla** | 1 | 1.9508 | 0.1625 |
| **comor12** | 1 | 0.0179 | 0.8934 |
| **any_use_of_nephrtoxi** | 1 | 0.0506 | 0.8221 |
| **procalcitonin_upon_a** | 1 | 0.0015 | 0.9693 |
| **cultures** | 1 | 0.1689 | 0.6811 |
| **baseline_creatinin** | 1 | 0.0058 | 0.9395 |
| **Srcr_24_hour_post_ad** | 1 | 1.2105 | 0.2712 |
| **acute_kidney_injury_** | 1 | 0.0255 | 0.8732 |
| **Vancomycin** | 1 | 0.0118 | 0.9136 |
| **lactic_acid_updated** | 1 | 2.0411 | 0.1531 |
| **Aminoglycosides** | 1 | 1.4003 | 0.2367 |
| **beta_lactam_choice** | 1 | 1.2791 | 0.2581 |
| **escalation_of_therap** | 1 | 1.7088 | 0.1911 |
| **institute** | 1 | 1.3943 | 0.2377 |

| **Note:** | No (additional) effects met the 0.1 significance level for entry into the model. |
| --- | --- |

| **Summary of Forward Selection** | | | | | | |
| --- | --- | --- | --- | --- | --- | --- |
| **Step** | **Effect Entered** | **DF** | **Number In** | **Score Chi-Square** | **Pr > ChiSq** | **Variable Label** |
| **1** | **SrCr_48_hour_post_ad** | 1 | 1 | 14.9209 | 0.0001 | cr_48_hour_post_admission |
| **2** | **Respiratory_infectio** | 1 | 2 | 9.8245 | 0.0017 | infect1 |
| **3** | **age** | 1 | 3 | 11.7260 | 0.0006 | age |
| **4** | **other_nephrotoxic_dr** | 1 | 4 | 6.3117 | 0.0120 | nephrotoxic_drug___5 |
| **5** | **corticosteroids** | 1 | 5 | 5.6911 | 0.0171 | corticostero |
| **6** | **vasopressors** | 1 | 6 | 4.8817 | 0.0271 | vasopressors |
| **7** | **Upon_enrollment_SrCr** | 1 | 7 | 5.1120 | 0.0238 | lab_upon_admission_cr |
| **8** | **comor3** | 1 | 8 | 2.7980 | 0.0944 | comor3 |

| **Partition for the Hosmer and Lemeshow Test** | | | | | |
| --- | --- | --- | --- | --- | --- |
| **Group** | **Total** | **late_ab = 1** | | **late_ab = 0** | |
|  |  | **Observed** | **Expected** | **Observed** | **Expected** |
| **1** | 22 | 3 | 2.47 | 19 | 19.53 |
| **2** | 22 | 3 | 7.65 | 19 | 14.35 |
| **3** | 22 | 8 | 10.30 | 14 | 11.70 |
| **4** | 22 | 18 | 12.06 | 4 | 9.94 |
| **5** | 22 | 17 | 13.78 | 5 | 8.22 |
| **6** | 22 | 15 | 15.68 | 7 | 6.32 |
| **7** | 22 | 17 | 16.80 | 5 | 5.20 |
| **8** | 22 | 17 | 17.97 | 5 | 4.03 |
| **9** | 22 | 19 | 19.06 | 3 | 2.94 |
| **10** | 26 | 23 | 24.23 | 3 | 1.77 |

| **Hosmer and Lemeshow Goodness-of-Fit Test** | | |
| --- | --- | --- |
| **Chi-Square** | **DF** | **Pr > ChiSq** |
| 15.2337 | 8 | 0.0548 |

| **Model Information** | | |
| --- | --- | --- |
| **Data Set** | WORK.TOSAS1 |  |
| **Response Variable** | late_ab | late_ab |
| **Number of Response Levels** | 2 |  |
| **Model** | binary logit |  |
| **Optimization Technique** | Fisher's scoring |  |

| **Number of Observations Read** | 224 |
| --- | --- |
| **Number of Observations Used** | 224 |

| **Response Profile** | | |
| --- | --- | --- |
| **Ordered Value** | **late_ab** | **Total Frequency** |
| **1** | 0 | 84 |
| **2** | 1 | 140 |

| ***Probability modeled is late_ab=1.*** |
| --- |

| **Model Convergence Status** |
| --- |
| Convergence criterion (GCONV=1E-8) satisfied. |

| **Model Fit Statistics** | | |
| --- | --- | --- |
| **Criterion** | **Intercept Only** | **Intercept and Covariates** |
| **AIC** | 298.380 | 257.278 |
| **SC** | 301.792 | 281.159 |
| **-2 Log L** | 296.380 | 243.278 |

| **Testing Global Null Hypothesis: BETA=0** | | | |
| --- | --- | --- | --- |
| **Test** | **Chi-Square** | **DF** | **Pr > ChiSq** |
| **Likelihood Ratio** | 53.1026 | 6 | <.0001 |
| **Score** | 47.9506 | 6 | <.0001 |
| **Wald** | 36.8908 | 6 | <.0001 |

| **Analysis of Maximum Likelihood Estimates** | | | | | |
| --- | --- | --- | --- | --- | --- |
| **Parameter** | **DF** | **Estimate** | **Standard Error** | **Wald Chi-Square** | **Pr > ChiSq** |
| **Intercept** | 1 | 4.2206 | 0.9047 | 21.7658 | <.0001 |
| **Upon_enrollment_SrCr** | 1 | -0.00596 | 0.00167 | 12.8108 | 0.0003 |
| **age** | 1 | -0.0385 | 0.0106 | 13.2407 | 0.0003 |
| **Respiratory_infectio** | 1 | 1.0814 | 0.3236 | 11.1699 | 0.0008 |
| **comor3** | 1 | -1.1051 | 0.7034 | 2.4684 | 0.1162 |
| **vasopressors** | 1 | -1.4721 | 0.5524 | 7.1014 | 0.0077 |
| **corticosteroids** | 1 | 1.3129 | 0.3721 | 12.4455 | 0.0004 |

| **Odds Ratio Estimates** | | | |
| --- | --- | --- | --- |
| **Effect** | **Point Estimate** | **95% Wald Confidence Limits** | |
| **Upon_enrollment_SrCr** | 0.994 | 0.991 | 0.997 |
| **age** | 0.962 | 0.942 | 0.982 |
| **Respiratory_infectio** | 2.949 | 1.564 | 5.560 |
| **comor3** | 0.331 | 0.083 | 1.315 |
| **vasopressors** | 0.229 | 0.078 | 0.677 |
| **corticosteroids** | 3.717 | 1.792 | 7.708 |

| **Association of Predicted Probabilities and Observed Responses** | | | |
| --- | --- | --- | --- |
| **Percent Concordant** | 77.3 | **Somers' D** | 0.546 |
| **Percent Discordant** | 22.7 | **Gamma** | 0.546 |
| **Percent Tied** | 0.0 | **Tau-a** | 0.257 |
| **Pairs** | 11760 | **c** | 0.773 |

| **Partition for the Hosmer and Lemeshow Test** | | | | | |
| --- | --- | --- | --- | --- | --- |
| **Group** | **Total** | **late_ab = 1** | | **late_ab = 0** | |
|  |  | **Observed** | **Expected** | **Observed** | **Expected** |
| **1** | 22 | 5 | 3.85 | 17 | 18.15 |
| **2** | 22 | 3 | 8.04 | 19 | 13.96 |
| **3** | 22 | 8 | 10.48 | 14 | 11.52 |
| **4** | 22 | 17 | 11.73 | 5 | 10.27 |
| **5** | 22 | 17 | 13.51 | 5 | 8.49 |
| **6** | 22 | 16 | 15.23 | 6 | 6.77 |
| **7** | 22 | 16 | 16.62 | 6 | 5.38 |
| **8** | 22 | 16 | 17.83 | 6 | 4.17 |
| **9** | 22 | 18 | 18.75 | 4 | 3.25 |
| **10** | 26 | 24 | 23.95 | 2 | 2.05 |

| **Hosmer and Lemeshow Goodness-of-Fit Test** | | |
| --- | --- | --- |
| **Chi-Square** | **DF** | **Pr > ChiSq** |
| 15.3433 | 8 | 0.0528 |
