# Supplementary material for: Early Versus Late Antipseudomonal β-Lactam Antibiotic Dose Adjustment in Critically Ill Sepsis Patients With Acute Kidney Injury: A Prospective Observational Cohort Study
Source: Open Forum Infect Dis. 2024 Feb 1;11(3):ofae059. doi: 10.1093/ofid/ofae059 (PMC10906704; doi:10.1093/ofid/ofae059)
Supplement: ofae059_Supplementary_Data [file ofae059_supplementary_data.zip › ADDITI~1.RTF]

Data Information	
Data Set	WORK.TOSAS1	
Output Data Set	WORK.OUTEX1	
Treatment Variable	late_ab	
Treated Group	0	
All Obs (Treated)	84	
All Obs (Control)	140	
Support Region	All Obs	
Lower PS Support	0.03683	
Upper PS Support	0.957258	
Support Region Obs (Treated)	84	
Support Region Obs (Control)	140	


Propensity Score Information	
Observations	Treated (late_ab = 0)	
	N	Weight	Mean	Standard
Deviation	Minimum	Maximum	
All	84		0.5132	0.2349	0.0869	0.9573	
Region	84		0.5132	0.2349	0.0869	0.9573	
Weighted	84	225.69	0.3722	0.2296	0.0869	0.9573	

Propensity Score Information	
Observations	Control (late_ab = 1)	Treated -
Control	
	N	Weight	Mean	Standard
Deviation	Minimum	Maximum	Mean
Difference	
All	140		0.2921	0.1779	0.0368	0.8623	0.2211	
Region	140		0.2921	0.1779	0.0368	0.8623	0.2211	
Weighted	140	219.69	0.3627	0.2127	0.0368	0.8623	0.0095	

Variable Information	
Variable	Observations	Treated (late_ab = 0)	
		N	Weight	Mean	Standard
Deviation	Minimum	Maximum	
apache_score	All	84		25.2738	6.95658	10.00000	44.0000	
	Region	84		25.2738	6.95658	10.00000	44.0000	
	Weighted	84	225.69	24.5548	7.04320	10.00000	44.0000	
age	All	84		66.9438	14.56782	25.98876	94.1745	
	Region	84		66.9438	14.56782	25.98876	94.1745	
	Weighted	84	225.69	62.4513	16.01542	25.98876	94.1745	
sofa	All	84		9.6071	3.02606	3.00000	16.0000	
	Region	84		9.6071	3.02606	3.00000	16.0000	
	Weighted	84	225.69	9.5699	2.85373	3.00000	16.0000	
lactic_acid_updated	All	84		4.4157	2.95972	0.80000	15.0000	
	Region	84		4.4157	2.95972	0.80000	15.0000	
	Weighted	84	225.69	4.4849	3.05050	0.80000	15.0000	
Time_to_firstABX_mea	All	84		120.2790	82.66747	1.00000	360.0000	
	Region	84		120.2790	82.66747	1.00000	360.0000	
	Weighted	84	225.69	111.9604	79.57512	1.00000	360.0000	
Male	All	84		0.5595	0.49644			
	Region	84		0.5595	0.49644			
	Weighted	84	225.69	0.5762	0.49416			
any_use_of_nephrtoxi	All	84		0.1190	0.32384			
	Region	84		0.1190	0.32384			
	Weighted	84	225.69	0.1148	0.31876			
vasopressors	All	84		0.0833	0.27639			
	Region	84		0.0833	0.27639			
	Weighted	84	225.69	0.1344	0.34112			
corticosteroids	All	84		0.4405	0.49644			
	Region	84		0.4405	0.49644			
	Weighted	84	225.69	0.3716	0.48324			
Renal_Replacement_Th	All	84		0.7857	0.41033			
	Region	84		0.7857	0.41033			
	Weighted	84	225.69	0.8504	0.35672			
Hematologic_malignan	All	84		0.9524	0.21296			
	Region	84		0.9524	0.21296			
	Weighted	84	225.69	0.9433	0.23135			
Non_hematologic_mali	All	84		0.8095	0.39268			
	Region	84		0.8095	0.39268			
	Weighted	84	225.69	0.8162	0.38731			
Solid_organ_transpla	All	84		0.9881	0.10846			
	Region	84		0.9881	0.10846			
	Weighted	84	225.69	0.9702	0.16991			
Autoimmune_diseases	All	84		0.9762	0.15246			
	Region	84		0.9762	0.15246			
	Weighted	84	225.69	0.9643	0.18555			
Chronic_Kidney_Disea	All	84		0.8452	0.36168			
	Region	84		0.8452	0.36168			
	Weighted	84	225.69	0.8904	0.31239			
Cardiovascular	All	84		0.2143	0.41033			
	Region	84		0.2143	0.41033			
	Weighted	84	225.69	0.2352	0.42412			
Respiratory_infectio	All	84		0.6190	0.48562			
	Region	84		0.6190	0.48562			
	Weighted	84	225.69	0.4766	0.49945			
Intrabdominal_infect	All	84		0.7143	0.45175			
	Region	84		0.7143	0.45175			
	Weighted	84	225.69	0.7772	0.41611			
Urinary_tract_infect	All	84		0.7976	0.40177			
	Region	84		0.7976	0.40177			
	Weighted	84	225.69	0.8184	0.38548			
Skin_soft_tissue_inf	All	84		0.8690	0.33735			
	Region	84		0.8690	0.33735			
	Weighted	84	225.69	0.8950	0.30658			
escalation_of_therap	All	84		0.8452	0.36168			
	Region	84		0.8452	0.36168			
	Weighted	84	225.69	0.8714	0.33477			

Variable Information	
Variable	Observations	Control (late_ab = 1)	Treated -
Control	
		N	Weight	Mean	Standard
Deviation	Minimum	Maximum	Mean
Difference	
apache_score	All	140		22.7000	6.66322	7.00000	40.0000	2.5738	
	Region	140		22.7000	6.66322	7.00000	40.0000	2.5738	
	Weighted	140	219.69	23.1327	6.56097	7.00000	40.0000	1.4221	
age	All	140		60.1182	16.91812	23.01033	91.4898	6.8256	
	Region	140		60.1182	16.91812	23.01033	91.4898	6.8256	
	Weighted	140	219.69	62.1814	16.47864	23.01033	91.4898	0.2699	
sofa	All	140		8.8643	3.13305	2.00000	18.0000	0.7429	
	Region	140		8.8643	3.13305	2.00000	18.0000	0.7429	
	Weighted	140	219.69	8.9969	3.01290	2.00000	18.0000	0.5730	
lactic_acid_updated	All	140		3.6836	3.36682	0.60000	24.0000	0.7321	
	Region	140		3.6836	3.36682	0.60000	24.0000	0.7321	
	Weighted	140	219.69	3.6413	3.23100	0.60000	24.0000	0.8436	
Time_to_firstABX_mea	All	140		121.6246	81.97161	4.00000	360.0000	-1.3455	
	Region	140		121.6246	81.97161	4.00000	360.0000	-1.3455	
	Weighted	140	219.69	124.6013	84.48900	4.00000	360.0000	-12.6409	
Male	All	140		0.6571	0.47466			-0.0976	
	Region	140		0.6571	0.47466			-0.0976	
	Weighted	140	219.69	0.6680	0.47094			-0.0918	
any_use_of_nephrtoxi	All	140		0.1071	0.30929			0.0119	
	Region	140		0.1071	0.30929			0.0119	
	Weighted	140	219.69	0.0994	0.29916			0.0154	
vasopressors	All	140		0.1643	0.37053			-0.0810	
	Region	140		0.1643	0.37053			-0.0810	
	Weighted	140	219.69	0.1332	0.33977			0.0013	
corticosteroids	All	140		0.3286	0.46969			0.1119	
	Region	140		0.3286	0.46969			0.1119	
	Weighted	140	219.69	0.3771	0.48465			-0.0054	
Renal_Replacement_Th	All	140		0.8286	0.37688			-0.0429	
	Region	140		0.8286	0.37688			-0.0429	
	Weighted	140	219.69	0.8376	0.36882			0.0128	
Hematologic_malignan	All	140		0.9643	0.18558			-0.0119	
	Region	140		0.9643	0.18558			-0.0119	
	Weighted	140	219.69	0.9645	0.18515			-0.0212	
Non_hematologic_mali	All	140		0.8286	0.37688			-0.0190	
	Region	140		0.8286	0.37688			-0.0190	
	Weighted	140	219.69	0.8379	0.36851			-0.0217	
Solid_organ_transpla	All	140		0.9643	0.18558			0.0238	
	Region	140		0.9643	0.18558			0.0238	
	Weighted	140	219.69	0.9672	0.17816			0.0031	
Autoimmune_diseases	All	140		0.9643	0.18558			0.0119	
	Region	140		0.9643	0.18558			0.0119	
	Weighted	140	219.69	0.9699	0.17093			-0.0056	
Chronic_Kidney_Disea	All	140		0.9286	0.25754			-0.0833	
	Region	140		0.9286	0.25754			-0.0833	
	Weighted	140	219.69	0.8806	0.32421			0.0098	
Cardiovascular	All	140		0.3286	0.46969			-0.1143	
	Region	140		0.3286	0.46969			-0.1143	
	Weighted	140	219.69	0.2782	0.44811			-0.0430	
Respiratory_infectio	All	140		0.4143	0.49260			0.2048	
	Region	140		0.4143	0.49260			0.2048	
	Weighted	140	219.69	0.4795	0.49958			-0.0030	
Intrabdominal_infect	All	140		0.8214	0.38299			-0.1071	
	Region	140		0.8214	0.38299			-0.1071	
	Weighted	140	219.69	0.7992	0.40060			-0.0220	
Urinary_tract_infect	All	140		0.8214	0.38299			-0.0238	
	Region	140		0.8214	0.38299			-0.0238	
	Weighted	140	219.69	0.7951	0.40362			0.0233	
Skin_soft_tissue_inf	All	140		0.9286	0.25754			-0.0595	
	Region	140		0.9286	0.25754			-0.0595	
	Weighted	140	219.69	0.9284	0.25778			-0.0334	
escalation_of_therap	All	140		0.8071	0.39454			0.0381	
	Region	140		0.8071	0.39454			0.0381	
	Weighted	140	219.69	0.8014	0.39896			0.0700	


Standardized Mean Differences (Treated - Control)	
Variable	Observations	Mean
Difference	Standard
Deviation	Standardized
Difference	Percent
Reduction	Variance
Ratio	
apache_score	All	2.5738	6.81148	0.37786		1.0900	
	Region	2.5738		0.37786	0.00	1.0900	
	Weighted	1.4221		0.20878	44.75	1.1524	
age	All	6.8256	15.78677	0.43236		0.7415	
	Region	6.8256		0.43236	0.00	0.7415	
	Weighted	0.2699		0.01709	96.05	0.9446	
sofa	All	0.7429	3.08002	0.24119		0.9329	
	Region	0.7429		0.24119	0.00	0.9329	
	Weighted	0.5730		0.18605	22.86	0.8971	
lactic_acid_updated	All	0.7321	3.16981	0.23097		0.7728	
	Region	0.7321		0.23097	0.00	0.7728	
	Weighted	0.8436		0.26614	0.00	0.8914	
Time_to_firstABX_mea	All	-1.3455	82.32027	-0.01634		1.0171	
	Region	-1.3455		-0.01634	0.00	1.0171	
	Weighted	-12.6409		-0.15356	0.00	0.8871	
Male	All	-0.0976	0.48568	-0.20100		1.0939	
	Region	-0.0976		-0.20100	0.00	1.0939	
	Weighted	-0.0918		-0.18893	6.00	1.1010	
any_use_of_nephrtoxi	All	0.0119	0.31665	0.03760		1.0963	
	Region	0.0119		0.03760	0.00	1.0963	
	Weighted	0.0154		0.04867	0.00	1.1353	
vasopressors	All	-0.0810	0.32687	-0.24766		0.5564	
	Region	-0.0810		-0.24766	0.00	0.5564	
	Weighted	0.0013		0.00383	98.45	1.0079	
corticosteroids	All	0.1119	0.48325	0.23157		1.1171	
	Region	0.1119		0.23157	0.00	1.1171	
	Weighted	-0.0054		-0.01126	95.14	0.9942	
Renal_Replacement_Th	All	-0.0429	0.39396	-0.10879		1.1853	
	Region	-0.0429		-0.10879	0.00	1.1853	
	Weighted	0.0128		0.03239	70.22	0.9355	
Hematologic_malignan	All	-0.0119	0.19974	-0.05960		1.3169	
	Region	-0.0119		-0.05960	0.00	1.3169	
	Weighted	-0.0212		-0.10614	0.00	1.5613	
Non_hematologic_mali	All	-0.0190	0.38486	-0.04949		1.0856	
	Region	-0.0190		-0.04949	0.00	1.0856	
	Weighted	-0.0217		-0.05643	0.00	1.1046	
Solid_organ_transpla	All	0.0238	0.15199	0.15665		0.3416	
	Region	0.0238		0.15665	0.00	0.3416	
	Weighted	0.0031		0.02015	87.13	0.9095	
Autoimmune_diseases	All	0.0119	0.16983	0.07010		0.6749	
	Region	0.0119		0.07010	0.00	0.6749	
	Weighted	-0.0056		-0.03285	53.14	1.1784	
Chronic_Kidney_Disea	All	-0.0833	0.31396	-0.26543		1.9722	
	Region	-0.0833		-0.26543	0.00	1.9722	
	Weighted	0.0098		0.03108	88.29	0.9284	
Cardiovascular	All	-0.1143	0.44101	-0.25915		0.7632	
	Region	-0.1143		-0.25915	0.00	0.7632	
	Weighted	-0.0430		-0.09754	62.36	0.8958	
Respiratory_infectio	All	0.2048	0.48912	0.41863		0.9719	
	Region	0.2048		0.41863	0.00	0.9719	
	Weighted	-0.0030		-0.00607	98.55	0.9995	
Intrabdominal_infect	All	-0.1071	0.41879	-0.25584		1.3913	
	Region	-0.1071		-0.25584	0.00	1.3913	
	Weighted	-0.0220		-0.05247	79.49	1.0789	
Urinary_tract_infect	All	-0.0238	0.39250	-0.06066		1.1005	
	Region	-0.0238		-0.06066	0.00	1.1005	
	Weighted	0.0233		0.05944	2.01	0.9121	
Skin_soft_tissue_inf	All	-0.0595	0.30011	-0.19834		1.7158	
	Region	-0.0595		-0.19834	0.00	1.7158	
	Weighted	-0.0334		-0.11144	43.81	1.4144	
escalation_of_therap	All	0.0381	0.37847	0.10066		0.8403	
	Region	0.0381		0.10066	0.00	0.8403	
	Weighted	0.0700		0.18499	0.00	0.7041	
Standard deviation of All observations used to compute standardized differences	


Observations with Largest IPTW-ATE Weights	
Treated (late_ab = 0)	Control (late_ab = 1)	
Expected Weight = 2.6667	Expected Weight = 1.6	
Observation	id	Weight	Scaled
Weight	Observation	id	Weight	Scaled
Weight	
105	105	11.51	4.32	4	4	7.26	4.54	
183	183	9.28	3.48	43	43	4.61	2.88	
54	54	8.36	3.13	24	24	4.00	2.50	
116	116	6.77	2.54	146	146	3.64	2.28	
51	51	6.72	2.52	103	103	3.39	2.12	
107	107	6.35	2.38	3	3	3.29	2.06	


Obs	id	late_ab	Male	age	_PS_	_ATEWgt_	
1	1	0	1	52.5716	0.27981	3.57384	
2	2	1	2	73.7557	0.25697	1.34583	
3	3	1	2	54.4969	0.69619	3.29155	
4	4	1	1	77.8154	0.86231	7.26295	
5	5	0	2	81.4243	0.53196	1.87985	
6	6	1	1	47.7571	0.23211	1.30228	
7	7	1	2	42.8440	0.08597	1.09406	
8	8	0	2	63.8337	0.19747	5.06412	
9	9	1	1	23.9142	0.04483	1.04694	
10	10	1	2	54.7557	0.49339	1.97390	

Model Information	
Data Set	WORK.NEW		
Dependent Variable	hosplos_90	hosplos_90	
Censoring Variable	hospdeath_90	hospdeath_90	
Censoring Value(s)	0		
Weight Variable	_ATEWgt_	ATE weight	
Ties Handling	BRESLOW		


Number of Observations Read
Number of Observations Used	224
224	


Class Level Information	
Class	Value	Design Variables	
late_ab	0	0		
	1	1		
				
Male	1	0		
	2	1		
				
any_use_of_nephrtoxic_drugs	0	0		
	1	1		
				
vasopressors	0	0		
	1	1		
				
corticosteroids	0	0		
	1	1		
				
Renal_Replacement_Therapy	0	0		
	1	1		
				
acute_kidney_injury_upon_p	1	0	0	
	2	1	0	
	3	0	1	
				
Hematologic_malignancy	0	0		
	1	1		
				
Non_hematologic_malignancy	0	0		
	1	1		
				
Solid_organ_transplantation	0	0		
	1	1		
				
Autoimmune_diseases	0	0		
	1	1		
				
comor10	0	0		
	1	1		
				
Chronic_Kidney_Disease	0	0		
	1	1		
				
Respiratory_infection	0	0		
	1	1		
				
Intrabdominal_infection	0	0		
	1	1		
				
Urinary_tract_infection	0	0		
	1	1		
				
Skin_soft_tissue_infection	0	0		
	1	1		


Summary of the Number of Event and Censored Values	
Total	Event	Censored	Percent
Censored	
224	86	138	61.61	


Convergence Status	
Convergence criterion (GCONV=1E-8) satisfied.	


Model Fit Statistics	
Criterion	Without
Covariates	With
Covariates	
-2 LOG L	831.042	764.490	
AIC	831.042	810.490	
SBC	831.042	866.940	


Testing Global Null Hypothesis: BETA=0	
Test	Chi-Square	DF	Pr > ChiSq	
Likelihood Ratio	66.5517	23	<.0001	
Score	72.3707	23	<.0001	
Wald	63.2832	23	<.0001	


Type 3 Tests	
Effect	LR Statistics	
	DF	Chi-Square	Pr > ChiSq	
late_ab	1	5.2659	0.0217	
apache_score	1	11.6499	0.0006	
age	1	0.1345	0.7138	
Male	1	3.6394	0.0564	
any_use_of_nephrtoxi	1	0.2091	0.6475	
sofa	1	1.4566	0.2275	
lactic_acid_updated	1	1.0349	0.3090	
vasopressors	1	0.5703	0.4502	
corticosteroids	1	0.1231	0.7257	
Time_to_firstABX_mea	1	0.8275	0.3630	
Renal_Replacement_Th	1	0.1170	0.7323	
Hematologic_malignan	1	4.5555	0.0328	
Non_hematologic_mali	1	12.7427	0.0004	
Solid_organ_transpla	1	0.2115	0.6456	
Autoimmune_diseases	1	0.9280	0.3354	
comor10	1	2.0441	0.1528	
Chronic_Kidney_Disea	1	4.0542	0.0441	
Respiratory_infectio	1	0.4909	0.4835	
Intrabdominal_infect	1	1.4722	0.2250	
Urinary_tract_infect	1	0.2300	0.6315	
Skin_soft_tissue_inf	1	0.2368	0.6265	
acute_kidney_injury_	2	4.1077	0.1282	


Analysis of Maximum Likelihood Estimates	
Parameter		DF	Parameter
Estimate	Standard
Error	Chi-Square	Pr > ChiSq	Hazard
Ratio	95% Hazard Ratio Profile Likelihood Confidence Limits	
late_ab	1	1	-0.55577	0.24469	5.1590	0.0231	0.574	0.352	0.923	
apache_score		1	0.07342	0.02179	11.3580	0.0008	1.076	1.032	1.124	
age		1	0.00320	0.00874	0.1343	0.7140	1.003	0.986	1.021	
Male	2	1	-0.46751	0.24664	3.5930	0.0580	0.627	0.384	1.013	
any_use_of_nephrtoxi	1	1	0.22118	0.49466	0.1999	0.6548	1.248	0.513	3.690	
sofa		1	0.07166	0.05937	1.4568	0.2274	1.074	0.956	1.207	
lactic_acid_updated		1	0.03752	0.03628	1.0694	0.3011	1.038	0.965	1.113	
vasopressors	1	1	-0.39671	0.51115	0.6023	0.4377	0.673	0.261	1.989	
corticosteroids	1	1	0.09719	0.27815	0.1221	0.7268	1.102	0.646	1.931	
Time_to_firstABX_mea		1	0.00136	0.00148	0.8436	0.3584	1.001	0.998	1.004	
Renal_Replacement_Th	1	1	-0.10076	0.29593	0.1159	0.7335	0.904	0.498	1.595	
Hematologic_malignan	1	1	1.10931	0.47565	5.4390	0.0197	3.032	1.104	7.304	
Non_hematologic_mali	1	1	1.14219	0.30985	13.5889	0.0002	3.134	1.691	5.712	
Solid_organ_transpla	1	1	-0.28365	0.63050	0.2024	0.6528	0.753	0.190	2.337	
Autoimmune_diseases	1	1	0.83826	0.80258	1.0909	0.2963	2.312	0.350	9.313	
comor10	1	1	0.41223	0.28631	2.0730	0.1499	1.510	0.857	2.639	
Chronic_Kidney_Disea	1	1	0.84205	0.39290	4.5932	0.0321	2.321	1.024	4.835	
Respiratory_infectio	1	1	-0.26055	0.37047	0.4946	0.4819	0.771	0.376	1.605	
Intrabdominal_infect	1	1	-0.50224	0.41920	1.4354	0.2309	0.605	0.261	1.354	
Urinary_tract_infect	1	1	-0.18728	0.39205	0.2282	0.6329	0.829	0.378	1.770	
Skin_soft_tissue_inf	1	1	0.21581	0.44141	0.2390	0.6249	1.241	0.512	2.914	
acute_kidney_injury_	2	1	-0.37188	0.33961	1.1990	0.2735	0.689	0.356	1.358	
acute_kidney_injury_	3	1	0.17461	0.35683	0.2395	0.6246	1.191	0.593	2.418	

Analysis of Maximum Likelihood Estimates	
Parameter		Label	
late_ab	1	late_ab 1	
apache_score		apache_score	
age		age	
Male	2	gendar 2	
any_use_of_nephrtoxi	1	any_nephro_drug 1	
sofa		sofa	
lactic_acid_updated		lactic_acid_updated	
vasopressors	1	vasopressors 1	
corticosteroids	1	corticostero 1	
Time_to_firstABX_mea		Time_to_firstABX_mean_impute	
Renal_Replacement_Th	1	rrt 1	
Hematologic_malignan	1	comor4 1	
Non_hematologic_mali	1	comor5 1	
Solid_organ_transpla	1	comor6 1	
Autoimmune_diseases	1	comor9 1	
comor10	1	comor10 1	
Chronic_Kidney_Disea	1	comor11 1	
Respiratory_infectio	1	infect1 1	
Intrabdominal_infect	1	infect2 1	
Urinary_tract_infect	1	infect3 1	
Skin_soft_tissue_inf	1	infect4 1	
acute_kidney_injury_	2	acute_kidney_injury_upon_p 2	
acute_kidney_injury_	3	acute_kidney_injury_upon_p 3	


Predictive Inaccuracy and Explained Variation	
Predictive Inaccuracy
(Smaller is Better)	Percent Explained Variation	
Without Covariates	With Covariates		
0.3740	0.2812	24.81	
