# Supplementary material for: Early Versus Late Antipseudomonal β-Lactam Antibiotic Dose Adjustment in Critically Ill Sepsis Patients With Acute Kidney Injury: A Prospective Observational Cohort Study
Source: Open Forum Infect Dis. 2024 Feb 1;11(3):ofae059. doi: 10.1093/ofid/ofae059 (PMC10906704; doi:10.1093/ofid/ofae059)

**Supplemental Digital Content**

**Early versus Late Antipseudomonal β-Lactam Antibiotic Dose Adjustment in Critically Ill Sepsis Patients with Acute Kidney Injury: A Prospective Observational Cohort Study**

**Table of Contents**

| Contents | Page number |
| --- | --- |
| Table S1: Institution Demographics | **3** |
| Table S2: Definitions | **4** |
| Table S3: Standardized Antipseudomonal β-Lactams Dosing | **5** |
| Figure S1: Time-Dependent Area Under the Curve for the main model | **6** |
| Figure S2: Model fit using Cox-Snell Residuals and Nelson-Aalen approach | **7** |

**Table S1: Brief Description of the Centers in Saudi Arabia**

| **Hospital name** | **Hospital Scope** | **Number of ICU Beds** | **Number of Patients Included** | **Type of ICUs** |
| --- | --- | --- | --- | --- |
| King Saud University Medical City- Riyadh (KSUMC) | Tertiary care  /Academic Medical Center | 48 | 77 | Mixed ICUs |
| King Faisal Specialist Hospital and Research Centre-Jeddah (KFSHRC-J) | Tertiary care | 23 | 32 | Medical ICU  Surgical ICU |
| Prince Sultan Military Medical City-Riyadh (PSMMC-R) | Tertiary care | 100 | 68 | Medical ICU  Surgical ICU  Trauma ICU  ED ICU |
| King Fahad Hospital-Madina  (KFH-M) | Tertiary care | 68 | 47 | Mixed ICUs |

**Table S2: Definitions**

| Sepsis  (Sepsis-3) | Organ dysfunction was identified as an acute change in total SOFA score by 2 points as a consequence of the infection |
| --- | --- |
| **Septic Shock**  **(Sepsis-3)** | Sepsis with persisting hypotension requiring vasopressors to maintain mean arterial pressure of 65 mm Hg & a serum lactate level >2 mmol/L (18 mg/dL) despite adequate volume resuscitation |
| **Time Zero**  **(Sepsis Recognition)** | The time from the earliest chart annotation is consistent with all elements of sepsis, including an alteration in mental status, systolic blood pressure ≤ 100 or/ and respiratory rate ≥ 22 |
| **Early β-lactam**  **Antibiotic**  **(E-BLA) dose adjustment** | Adjustment of β-lactam antibiotics within the first 24 hours of sepsis recognition |
| **Late β-lactam**  **Antibiotic**  **(L- BLA) dose adjustment** | Adjustment of β-lactams antibiotics after the first 24 hours of sepsis recognition |
| **Acute kidney injury (AKI)**  **(KIDIGO)** | Stage I: Increase in serum creatinine (SCr) 1.5 to 1.9 fold from baseline  Stage II: Increase in SCr 2-2.9 fold from baseline  Stage III: Increase in SCr 3 fold from baseline or SCr of ≥353.6 mmol/l  (Patients on stage III should not start on renal replacement therapy within the first 48 hours of sepsis recognition) |
| **Improvement of Renal function within 48 hours (transient AKI)** | Complete reversal of AKI by KDIGO criteria within 48 hours of AKI onset |
| **Resolution of kidney Injury (recovering of AKI at admission)** | Decrease in serum creatinine of > 50% occurring within seven days of AKI onset |
| **Escalation of antibiotic therapy** | To switch antibiotics with a lower spectrum of activity to antibiotics with a broader spectrum of activity.  Lower spectrum to higher spectrum antipseudomonal was defined as the following  Cefepime/ ceftazidime / piperacillin- tazobactam🡪 Carbapenem 🡪 ceftazidime – avibactam/ ceftolozane -tazobactam |

**Table S3: Standardized Antipseudomonal β-Lactams Dosing***

| **Antibiotic** | **Regimens Without Adjustment** | **Renally-Adjusted Regimens*** | | |
| --- | --- | --- | --- | --- |
|  |  | **CrCl** | **Dose** | **Frequency** |
| **Cefepime** | **First dose** = 2 g  **Maintenance dose =**   - 2 gm IV every 8 hours - 2 gm IV every 12 hours | 30-60 | 1 g | Every 12 hours |
|  |  | 11-29 | 2 g | Every 24 hours |
|  |  | <11 | 500 | Every 24 hours |
| **Ceftazidime** | **First Dose** = 2 g  **Maintenance dose =**   - 1 gm IV every 8 hours - 2 gm IV every 8 hours | 10-50 | 1-2 g | Every 12 hours |
|  |  | 10-25 | 1-2 g | Every 24 hours |
| **Imipenem- cilastatin** | **First dose** = 500 – 1 g  **Maintenance dose =**   - 500 mg IV every 6 hours - 1 g IV every 8 hours - 1 g IV every 6 hours | 60 - <90 | 400  or  750 mg | Every 6 hours  or  Every 8 hours |
|  |  | 30 - <60 | 500 mg  or  300 mg | Every 8 hours  or  Every 6 hours |
|  |  | 15 - <30 | 500 mg  or  200 mg | Every 12 hours  or  Every 6 hours |
| **Meropenem** | **First dose** **=** 1 g  **Maintenance dose =**   - 1 g IV every 8 hours | 26-50 | 1 g | Every 12 hours |
|  |  | 10-25 | 500 mg | Every 12 hours |
|  |  | < 10 | 500 mg | Every 24 hours |
| **Piperacillin/ Tazobactam** | **First dose** = 3.375 – 4.5 g  **Maintenance dose =**   - 3.375 IV every 6 hours - 4.5 IV every 6 hours | 20-40 | 3.375 g  or  2.25 g | Every 8 hours  or  Every 6 hours |
|  |  | < 20 | 2.25 g | Every 8 hours |

***** The doses are generally not specific to any indication

**Figure S1: Time-Dependent Area Under the Curve for the main model**

**
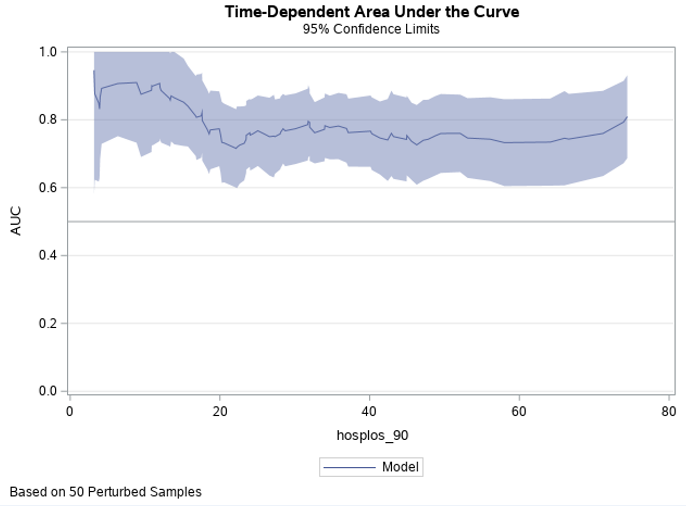
**

**Figure S2: Model fit using Cox-Snell Residuals and Nelson-Aalen approach**


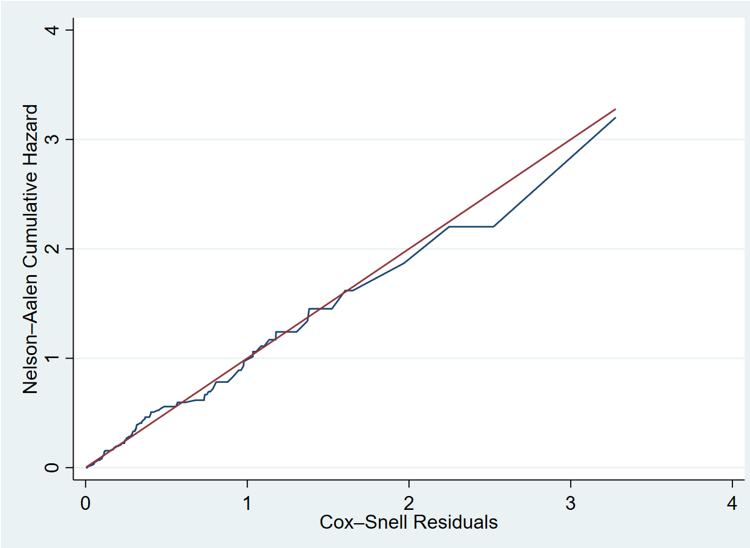

Supplement: ofae059_Supplementary_Data [file ofae059_supplementary_data.zip › Supplemental Digital Content.docx]
